# Structure and function of the ovipositor of the encyrtid wasp *Microterys flavus*

**DOI:** 10.1186/s12983-025-00575-1

**Published:** 2025-08-25

**Authors:** Robin Kraft, Oliver Betz, Alexander Rack, Benjamin Eggs

**Affiliations:** 1 Evolutionary Biology of Invertebrates, Institute of Evolution and Ecology, University of Tübingen, Auf der Morgenstelle 28, 72076 Tübingen, Germany; 2 ESRF – The European Synchrotron, Structure of Materials Group – ID19, CS 40220, 38043 Grenoble Cedex 9, France; 3 Department of Evolutionary Biology and Environmental Studies, University of Zurich, Winterthurerstrasse 190, 8057 Zurich, Switzerland

**Keywords:** Chalcidoidea, Encyrtidae, Functional morphology, Hymenoptera, Ovipositor, Parasitoid, Terebra

## Abstract

**Background:**

Oviposition is crucial for the reproductive success of parasitoid insects and, hence, ovipositor structure and oviposition behaviour have probably played a central role in their adaptive evolution. However, various mechanical and functional aspects of the musculoskeletal ovipositor system are still not fully understood, especially within the enormously diverse parasitoid wasps, e.g. the minute and understudied Encyrtidae (Chalcidoidea). Some encyrtid wasps are specialized in parasitising insect plant pests and thus play an important ecological and economic role. We have examined all inherent cuticular elements and muscles of the ovipositor of the encyrtid wasp *Microterys flavus* to improve our understanding of its mechanics and mode of function. We provide a detailed 3D model based on a synchrotron X-ray phase-contrast microtomography (SR-µCT) dataset and have analysed microstructures on the cuticular ovipositor elements by using scanning electron microscopy (SEM). We have also conducted an in vivo documentation of the oviposition process of female *M. flavus* wasps on their host, the scale insect *Coccus hesperidum*.

**Results:**

Based on morphological analyses, we have identified all elements of the musculoskeletal ovipositor system in *M. flavus*, consisting of two pairs of valvifers, three pairs of valvulae, the female T9 (9th abdominal tergum), and a set of nine paired ovipositor muscles. Three of these muscles (1st valvifer-genital membrane muscle, ventral 2nd valvifer-venom gland reservoir muscle, T9-genital membrane muscle) have only recently been discovered in pteromalid wasps but have not yet been described for encyrtids. Our behavioural analysis of the motion patterns during the various phases of parasitization has elucidated the oviposition process, which consists of penetration of the host’s body, assessment of the host’s internal organs, envenomation, egg deposition, and potential host feeding.

**Conclusions:**

Based on our studies of the structure of the ovipositor system of the encyrtid wasp *M. flavus*, we have developed a functional model of the underlying working mechanism of all ovipositor movements observed during the oviposition process, thereby improving our understanding of a possible key trait contributing to the evolutionary success of a highly diverse group of chalcidoid wasps.

**Supplementary Information:**

The online version contains supplementary material available at 10.1186/s12983-025-00575-1.

## Background

Hymenopterans are a highly diverse group of insects, currently including more than 150,000 described species [1]. This diversification was mostly driven by the development of parasitoidism, with about 70% of today’s hymenopteran insects living a parasitoid life cycle [2]. Nevertheless, this diversification is not only associated with a single trait but has also been driven by a combination of a set of key innovations, one of them being the development of a constriction between the 1st and the 2nd abdominal segment, the wasp waist, leading to the evolution of the apocritan wasps (suborder Apocrita) [2, 3]. This morphological innovation greatly improved the manoeuvrability of the abdomen and thereby made a more precise employment of the female ovipositor possible [1–3]. In addition to the deposition of eggs, the ovipositor was then capable of precisely penetrating and accessing the host, of drilling through the substrate or a puparium, of evaluating the suitability of the host, or of injecting venom [4, 5]. Although the basic structure of the ovipositor is similar across all parasitoid wasps, and even across all hymenopterans [6, 7], numerous modifications and variations have been made [8–10] that putatively have laid the morphological foundation for niche subdivision through host specialization, which has in turn increased speciation rates and led to the rapid diversification of parasitoid wasps [1, 2, 11–13].

**Fig. 1:**
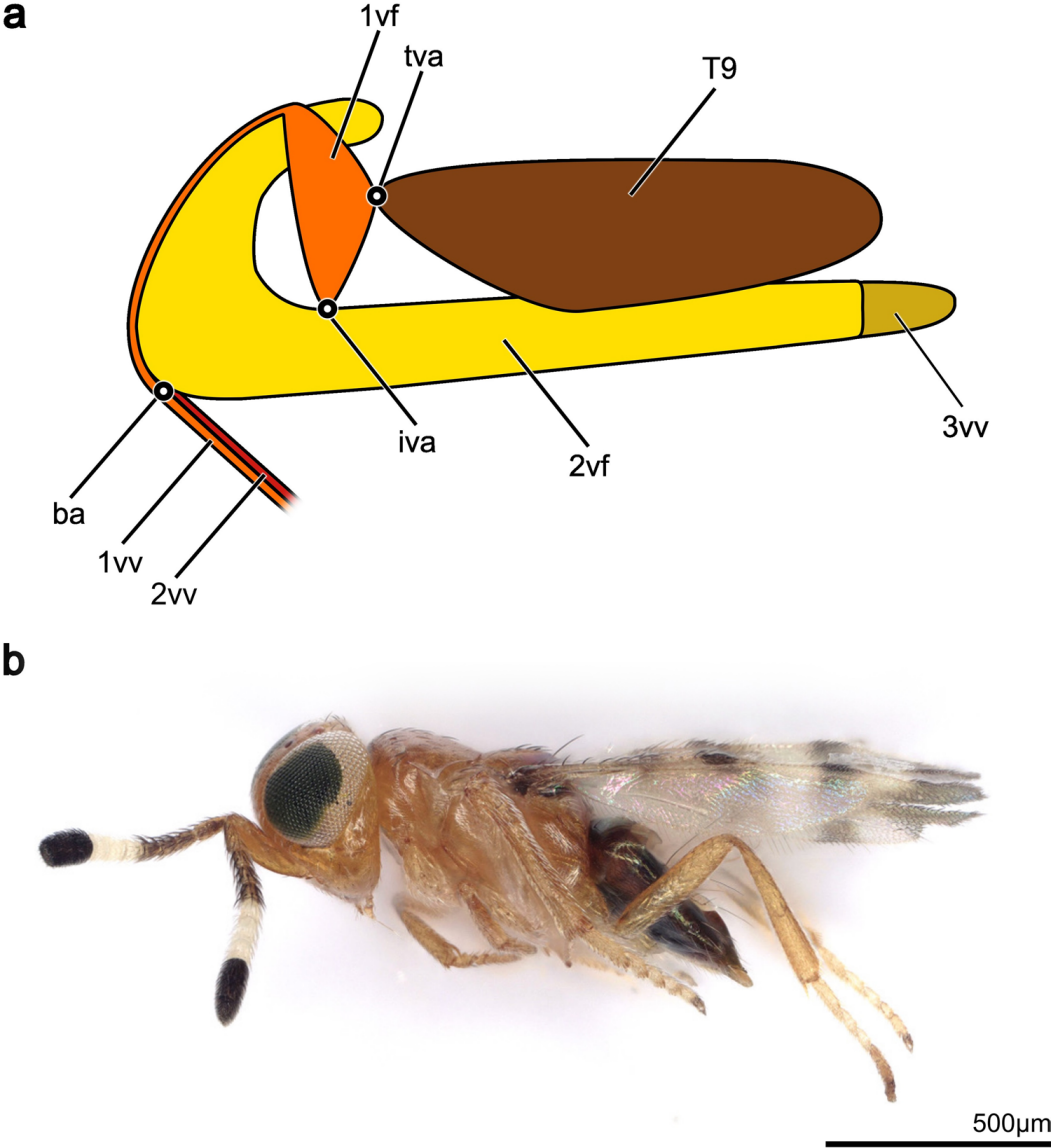
**a** Schematic representation of the hymenopteran ovipositor (lateral view, left is anterior). All elements appear pairwise except for the female T9 and the 2nd valvula. The 1st valvulae and 2nd valvula form the terebra. **b** Habitus image of a female *Microterys flavus* (lateral view). *Abbreviations*: 1vf: 1st valvifer; 1vv: 1st valvula; 2vf: 2nd valvifer; 2vv: 2nd valvula; 3vv: 3rd valvula; ba: Basal articulation; iva: Intervalvifer articulation; T9: Female T9; tva: Tergo-valvifer articulation

Generally, the hymenopteran ovipositor is an anatomical cluster of multiple cuticular structures that are actuated by a set of muscles. All the involved structures derive from the 8th and 9th abdominal segments (= 7th and 8th metasomal segments) of the female insect [6, 10]. The following structures are part of the hymenopteran ovipositor groundplan: the 1st valvifers (1vf; Fig. 1a), the 2nd valvifers (2vf; Fig. 1a), the 1st valvulae (1vv; Fig. 1a), the 2nd valvula (2vv; Fig. 1a), the 3rd valvulae (3vv; Fig. 1a), and the female T9 (T9; Fig. 1a) (for the homologies and the synonyms most commonly found in the literature, see Table 1). All the cuticular structures mentioned above appear pairwise except for the female T9 and the 2nd valvula, with the latter being a fusion of the 9th gonapophyses. However, the 2nd valvula is secondarily separated except at the base and the apex in many representatives of parasitoid wasps [8]. The basal elements of the ovipositor bear the muscles actuating the ovipositor movement, whereas the terebra (= ovipositor shaft sensu [5]) lacks any intrinsic musculature [6, 10]. The whole ovipositor system is attached to the metasoma through the female T9, which extends dorsally beneath the other tergites. The 2nd valvifers are elongated and their posterior parts are situated medially of the female T9. The 1st valvifers lie between the female T9 and the 2nd valvifers and are connected to both via two articulations. Their posterior angles are connected to the female T9 via the tergo-valvifer articulation (tva; Fig. 1a) and ventrally to the 2nd valvifer through the intervalvifer articulation (iva; Fig. 1a). Each of the 1st valvifers extends dorsally into a dorsal ramus, which runs anterodorsally around the edge of the 2nd valvifer and is therefore continuous with the 1st valvula. Together with the 2nd valvula, the paired 1st valvulae form the terebra. More precisely, the terebra comprises three elements: a pair of ventrally located 1st valvulae and the 2nd valvula, forming the dorsal element. Proximally, the 2nd valvula has lateral basal thickenings called the bulbs. Here, the 2nd valvula is articulated with the anteroventral part of the 2nd valvifer via the basal articulation (ba; Fig. 1a) [6, 10]. The 1st and 2nd valvulae are interconnected via a longitudinal tongue-and-grove-like interlocking mechanism called the olistheter [8, 10]. Both the 1st and 2nd valvulae, connected through the olistheter, form the egg canal that transports the egg along the terebra into the host’s body, often also delivering venom or other secretions [7]. The pair of 3rd valvulae originates at the posterior end of the 2nd valvifers and embraces the terebra in the resting position.

**Table 1:** Morphological terms of the elements of the hymenopteran ovipositor according to the Hymenoptera Anatomy Ontology (HAO) [37–39], which we applied in the present study

Anatomical term (abbreviation)	Homology (cf. [6, 10])	Term used by Copland and King [18–22, 25] and others	Term used by Quicke [5, 7]
1st valvulae (1vv)	8th gonapophyses	Stylets [17–25]	Lower valves
2nd valvula (2vv)	Fusion of the 9th gonapophyses	(Stylet) sheath [17–25]	Upper valve
3rd valvulae (3vv)	9th gonostyli	Palps [18, 19, 21, 22]	Ovipositor sheaths
1st valvifers (1vf)	8th gonocoxites [14, 15] (fused with the gonangula [16])	Fulcral plates [17–25]	Gonangula
2nd valvifers (2vf)	9th gonocoxites	Inner ovipositor plates [17–20, 22–25]	–
Female T9 (T9)	9th abdominal tergum	Outer ovipositor plate [17–20, 22–25]	–

Homologies and the most common synonyms found in the literature on the chalcidoid ovipositor system (e.g. Copland and King [18–22, 25]) and the hymenopteran ovipositor in general (e.g. Quicke [5, 7]) are given

One of the most diverse groups of parasitoid hymenopterans is the superfamily Chalcidoidea. They have major ecological and economic importance, for instance, in the field of natural and agricultural plant pest control (e.g. [29–31]). Despite multiple studies on the ovipositor structure of representatives of various chalcidoid families (e.g. Agaonidae [25], Aphelinidae [17], Chalcididae [21, 26], Eulophidae [19], Eurytomidae [20, 27], Pteromalidae [22, 24, 32], Torymidae [18]), insights into their ecology, morphology or life-history remain limited. Indeed, for the Encyrtidae, which includes the species *Microterys flavus* (Howard, 1881) studied here, knowledge in this regard is completely lacking.

In the present study, we have investigated the oviposition behaviour and the structural and functional morphology of the musculoskeletal ovipositor system of *Microterys flavus* (Chalcidoidea: Encyrtidae) (Fig. 1b), a cosmopolitan (probably originally native to Southeast Asia or Pakistan [33]) solitary synanthropic idiobiont endoparasitoid of various soft scale insects (Coccidae), with special preference for the brown soft scale *Coccus hesperidum* Linnaeus, 1758 [34]. As an idiobiont endoparasitoid, *M. flavus* is an exception, as the vast majority of idiobionts are ectoparasitoids. *C. hesperidum* itself is a cosmopolitan parasite of a wide range of plant species and thus represents an ecological and economic burden to agricultural systems. The effect of *M. flavus* as a biological plant pest control has often been tested [34–36] and illustrates the ecological importance of this species. We have aimed (1) to analyse the oviposition process in vivo with special attention being paid to the employment of the terebra and (2) to describe the ovipositor of *M. flavus*, including all inherent cuticular elements and muscles, thus providing the first thorough description of the musculoskeletal ovipositor system of an encyrtid wasp.

## Results

We conducted high-resolution video recordings to analyse the behaviour of the wasps and combined this with morphological investigations based on microscopical (light microscopy, scanning electron microscopy) and microtomographical (synchrotron X-ray phase-contrast microtomography) techniques.

All morphological terms are applied in accordance with the Hymenoptera Anatomy Ontology (HAO; [37–39]; available online at http://glossary.hymao.org). All structures referred to in this work, their definitions, and the synonyms commonly found in the literature are given in Table 3 in Appendix 1.

### Behavioural analysis of oviposition behaviour

**Fig. 2:**
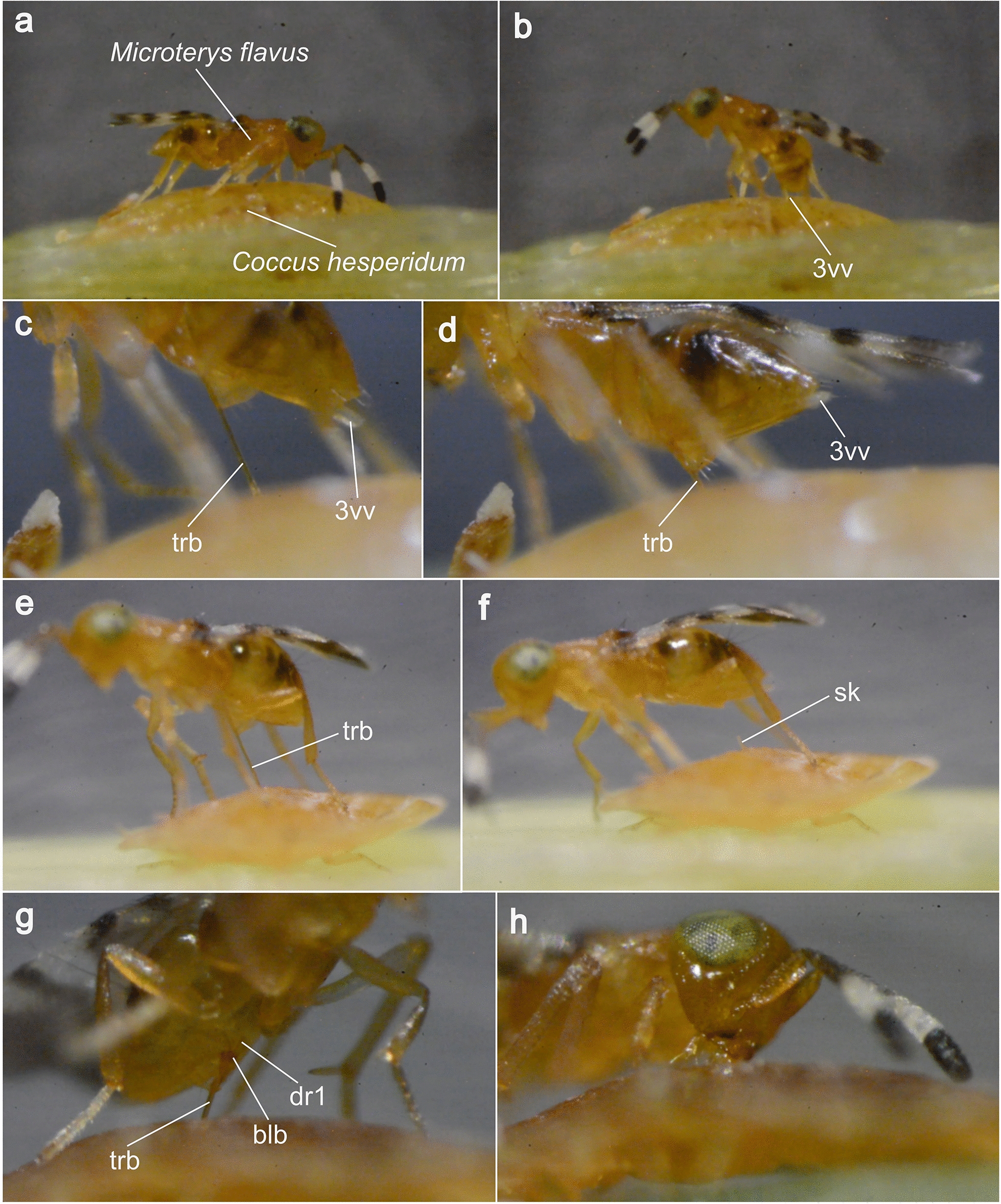
Single frames of high-resolution video recordings of the oviposition process of *Microterys flavus* on the host organism *Coccus hesperidum,* including host feeding. **a** Host finding: the female *M. flavus* scans the host’s dorsal shield by using its antenna. **b** Drilling: the metasoma is lowered to guide the terebra to the insertion site, also by using the sensory 3rd valvulae. **c** Drilling: the terebra is drilled through the host’s dorsal shield by using the ‘push–pull’ mechanism combined with rotational movements of the whole terebra. **d** Envenomation: with the terebra fully inserted, *M. flavus* performs rhythmic contractions of parts of the metasoma. **e, f** Egg deposition: with the terebra almost fully extracted, *M. flavus* performs trembling movements of the metasoma at a high frequency. After terebra extraction, a small stalk remains at the penetration site. **g, h** Host feeding: *M. flavus* accesses the host’s internal organs to reach body fluids. It feeds on leaking haemolymph by using its mouthparts. *Abbreviations*: 3vv: 3rd valvulae; blb: Bulbs; dr1: Dorsal ramus of the 1st valvula; sk: Stalk; trb: Terebra

We recorded the full oviposition act of *M. flavus* on its preferred host *C. hesperidum* (Fig. 2; Additional file 1), focusing on the employment of the terebra. Out of the five trials, two female wasps successfully completed oviposition, whereas two different individuals could be observed to feed on the host. No specimen was observed to perform both activities.

**Search for a potential host:** After both the host and its parasitoid were brought together into the experimental chamber, the female *M. flavus* needed only a few minutes to perceive the presence of the host and to determine its position. The wasp immediately started to skim the host’s dorsal shield intensely in multiple rounds, using both antenna (Fig. 2a; Additional file 1, min. 0:11–0:25).

**Penetration of the host’s skin:** After the wasp had found a suitable spot, it stretched its forelegs to raise its thorax upwards. Simultaneously, the metasoma was brought forward under the thorax, so that it was oriented perpendicularly to the dorsal shield of the host (Fig. 2b; Additional file 1, min. 0:25–0:29). In this position, the 3rd valvulae (3vv; Fig. 2b–d), still ensheathing the terebra, pointed downwards, directly towards the host. Subsequently, the metasoma, together with the 3rd valvulae, was lifted back to the initial position, with only the terebra remaining at the puncture site, the terebra having been released from the enclosure of the 3rd valvulae (Fig. 2c; Additional file 1, min. 0:30–0:34). The basal section of the terebra was now visibly enveloped by one of the abdominal sternites. In this position, the wasp started to access the host by drilling through the shield of the coccid, the female wasp frequently moving its entire body up and down. During this action, it additionally seemed to perform a behaviour that could be identified as a partial rotation of the terebra around its own axis (Additional file 1, min. 1:10–1:15). Apart from minor bending, the terebra remained in a straight and stiff posture during the drilling process. Because of the limited resolution and the constant movement of the whole terebra, movements of the individual valvulae were not recognisable. However, such movements probably take place. It took the wasp about 30 s to fully penetrate the dorsal cuticular shield (Additional file 1, min. 0:45–1:18).

**Assessment of host and envenomation:** After the penetration of the dorsal shield of the host, the wasp started to probe the inside of its body, using the full length of the terebra. To access all regions of the host’s body, the terebra was inserted, almost fully withdrawn, and then re-inserted multiple times (Additional file 1, min. 1:24–1:39). Throughout this process, the wasp constantly changed the position of its body and adjusted the insertion angle of the terebra. This process of active host assessment, between the terebra insertion and the initiation of egg deposition, took approximately one minute. However, the movement of the terebra inside the host’s body could not be observed, since it was concealed by the host’s tergum. Presumably, various areas and organs of the host body were being targeted at this stage.

**Egg deposition:** Having assessed the host, the female wasp remained in a fixed position, with the body lowered towards the host and the terebra fully inserted (Fig. 2d). Whereas the overall body movements were reduced during this process, the ventral area of the metasoma contracted at regular intervals (Additional file 1, min. 1:40–1:50). Having remained in this position for about one minute, the wasp slowly raised its entire body, subsequently withdrawing the terebra until only its apex remained beneath the host’s dorsal shield (Fig. 2e). This was followed by trembling movements of the metasoma at a very high frequency. Finally, under decreasing frequency, the wasp slowly withdrew the terebra completely from the host’s body (Additional file 1, min. 1:52–2:07). Once the terebra was fully removed from the host, only a small stalk remained at the penetration site, as a remnant of the oviposition (Fig. 2f). In total, the wasp needed approximately 90 s to conduct egg laying.

**Host feeding:** Two of the observed wasps performed host feeding but did not oviposit before or afterwards. Both females first had to gain access to the host’s internals to reach its body fluids for feeding. To do so, they carried out the previously described behaviours, including drilling, assessing the host, and probably also injecting venom (Fig. 2g; Additional file 1, min. 2:15–2:55). These processes were conducted within approximately four minutes. After the withdrawal of the terebra, the mouthparts were brought to the previously injured penetration site to feed on the haemolymph, which leaked out of the host’s wound (Fig. 2h; Additional file 1, min. 2:55–3:11). No feeding tube was detected at the injection site. In multiple rounds, the wasp then repeatedly penetrated the wound with its terebra, alternating with feeding at the site, although the duration of the cycles was significantly lower (on average 30 s; Additional file 1, min. 3:12–3:56). The first wasp repeated this cycle nine times, whereas the second one repeated it 15 times. Finally, the wasp finished the procedure with a long feeding period that lasted on average five minutes.

### Morphological analysis of the musculoskeletal ovipositor system

The musculoskeletal ovipositor system of *M. flavus* consists of three pairs of valvulae, two pairs of valvifers, the female T9, three paired articulations, and a set of nine paired muscles (Additional file 2). The structure of the ovipositor is bilateral, which means that all ovipositor elements and muscles are present as pairs, except for the 2nd valvula and the female T9. Nonetheless, in the following, all morphological structures are referred to in the singular form. All structures are given in Table 3 in Appendix 1. All associated muscles, their place of origin and insertion, and their assumed function are listed in Table 2.

**Table 2:** Ovipositor muscles of *Microterys flavus*. Muscle designation, abbreviation, morphological origin, and insertion in the ovipositor system and assumed functions

Muscle name (abbreviation)	Origin	Insertion	Assumed function
1st valvifer-genital membrane muscle (m-1vf-gm)	Medially at the posteroventral margin of the 1st valvifer, at the centre between the intervalvifer and the tergo-valvifer articulations (iva/tva) (Fig. 7d–f; 9c)	Anteriorly at the genital membrane (Fig. 7d–f; 9a)	Tensor muscle for stabilization of the 1st valvifers during ovipositor movements
Dorsal 2nd valvifer-venom gland reservoir muscle (m-d-2vf-vr)	Medial surface of the anterodorsal end of the 2nd valvifer (Fig. 7d–f; 8a, b; 10c)	Anterodorsal surface of the venom gland reservoir (Fig. 7d–f; 10a, b)	Aids in a controlled outflow of venom from the venom gland reservoir or a lubricant from the Dulfour’s gland into the terebra, presumably also expanding the common oviduct and thus aiding the controlled transfer of an egg into the egg canal of the terebra, further acting as a tensor muscle in stabilizing the 2nd valvifer during oviposition
Ventral 2nd valvifer-venom gland reservoir muscle part a (m-v-2vf-vr-a)	Medial surface of the anteroventral end of the 2nd valvifer, ventrally to m-2vf-vr (Fig. 7d–f; 8a, b; 10f)	Laterally at the orifice of the venom gland reservoir (Fig. 7d–f; 10d, e)	Controlling the discharge of venom by increasing the diameter of the orifice of the venom gland reservoir
Ventral 2nd valvifer- venom gland reservoir muscle part b (m-v-2vf-vr-b)	Medial surface of the anteroventral end of the 2nd valvifer, adjacent to the basal articulation (Fig. 7d–f; 8a, b; 10g)	Laterally at the orifice of the venom gland reservoir, shortly before the orifice of the venom gland reservoir enters the common oviduct, ventrally to the insertion of m-v-2vf-vr-a m-d-2vf-co (Fig. 7d–f; 10h, i)	
Anterior 2nd valvifer-2nd valvula muscle (m-a-2vf-2vv)	Medial surface of the anterodorsal arch of the 2nd valvifer (Fig. 7d–f; 8a, b)	At the processus articularis, laterally to the proximal bulbous end of the 2nd valvula (Fig. 7d–f; 8a)	Supporting the rotation of the terebra during drilling and oviposition, elevator of the terebra (towards the resting position) by dorsally rotating the bulb at the basal articulation, holding the terebra at resting position
Posterior 2nd valvifer-2nd valvula muscle (m-p-2vf-2vv)	Medial surface along the ventral part of the 2nd valvifer (Fig. 7d–f; 8a, b)	At the processus musculares, the anterodorsally directed processes of the bulbs of the 2nd valvula (Fig. 7d–f; 8a)	Rotation of the terebra and holding the terebra in active probing position during drilling and oviposition
Dorsal T9-2nd valvifer muscle part a (m-d-T9-2vf-a)	Along both the dorsolateral and dorsomedial surface of the female T9 along its medial ridge (Fig. 7d–f)	At the hook-shaped lobe of the 2nd valvifer along its dorsal flange (Fig. 7d–f)	Protractor of the 1st valvulae: moves the 2nd valvifer posteriorly and the female T9 anteriorly towards each other, causing the 1st valvifer to rotate anteriorly and, thus, the 1st valvula to slide distally relative to the 2nd valvula (work antagonistically to m-v-T9-2vf)
Dorsal T9-2nd valvifer muscle part b (m-d-T9-2vf-b)	Medial surface of the female T9 ventrally to the medial ridge of the T9 (Fig. 7d–f)	Anterior section of the dorsal flange of the 2nd valvifer, ventrally to the insertion of m-d-T9-2vf-a (Fig. 7d–f)	
Ventral T9-2nd valvifer muscle (m-v-T9-2vf)	At the cordate apodeme, situated around the anterior end of the female T9 (Fig. 7d–f)	Throughout almost the full posterior part of the dorsal flange of the 2nd valvifer (Fig. 7d–f)	Retractor of the 1st valvulae: Moves the 2nd valvifer anteriorly and the female T9 posteriorly apart from each other, causing the 1st valvifer to rotate posteriorly and, thus, the 1st valvula to slide proximally relative to the 2nd valvula (works antagonistically to m-d-T9-2vf a and b)
T9-genital membrane muscle (m-T9-gm)	Medial surface of the posterodorsal part of female T9, posterior to the origin of m-p-T9-2vf (Fig. 7d–f; 9d)	At the genital membrane between the 2nd valvifers (Fig. 7d–f; 9e)	Tensor muscles holding both the female T9 and the 2nd valvifer in their position during oviposition
Posterior T9-2nd valvifer muscle (m-p-T9-2vf)	Posterodorsal region of the female T9, anterior to the origin of the m-T9-gm (Fig. 7d–f; 9f, g)	At the median bridge connecting both posterodorsal ends of the 2nd valvifers (Fig. 7d–f; 9f)	Tensor muscles holding both the female T9 and the 2nd valvifer in their position during oviposition

All results were determined based on the SR-µCT dataset and the generated 3D model

#### Cuticular elements of the ovipositor

**Fig. 3:**
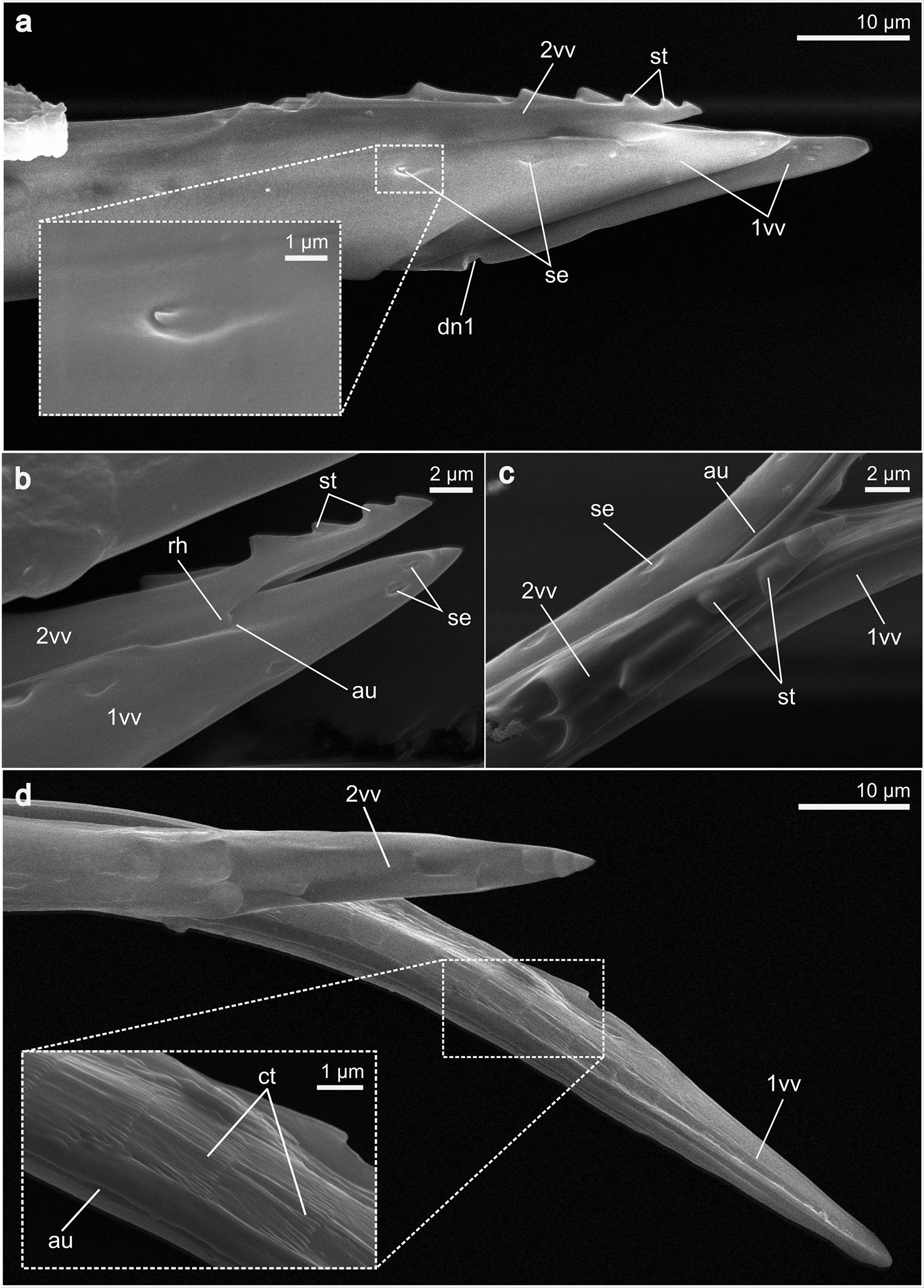
SEM images of the apex of the terebra *of Microterys flavus*. **a** Terebra apex (lateral view). Detailed aspect of sensilla. **b** Terebra apex (lateral view), showing the posterior end of the olistheter mechanism (comprising rhachis and aulax). **c** Detailed view of the apex of the 2nd valvula (dorsal aspect). Apex bears multiple sawteeth. **d** Dorsal aspect of the 1st valvulae around the apical area showing the terebra’s interior microstructures on the surface of the egg canal. *Abbreviations*: 1vv: 1st valvulae; 2vv: 2nd valvula; au: Aulax; dn1: Distal notch of the 1st valvula; se: Sensilla; ct: Ctenidia; rh: Rhachis; st: Sawteeth

**Fig. 4:**
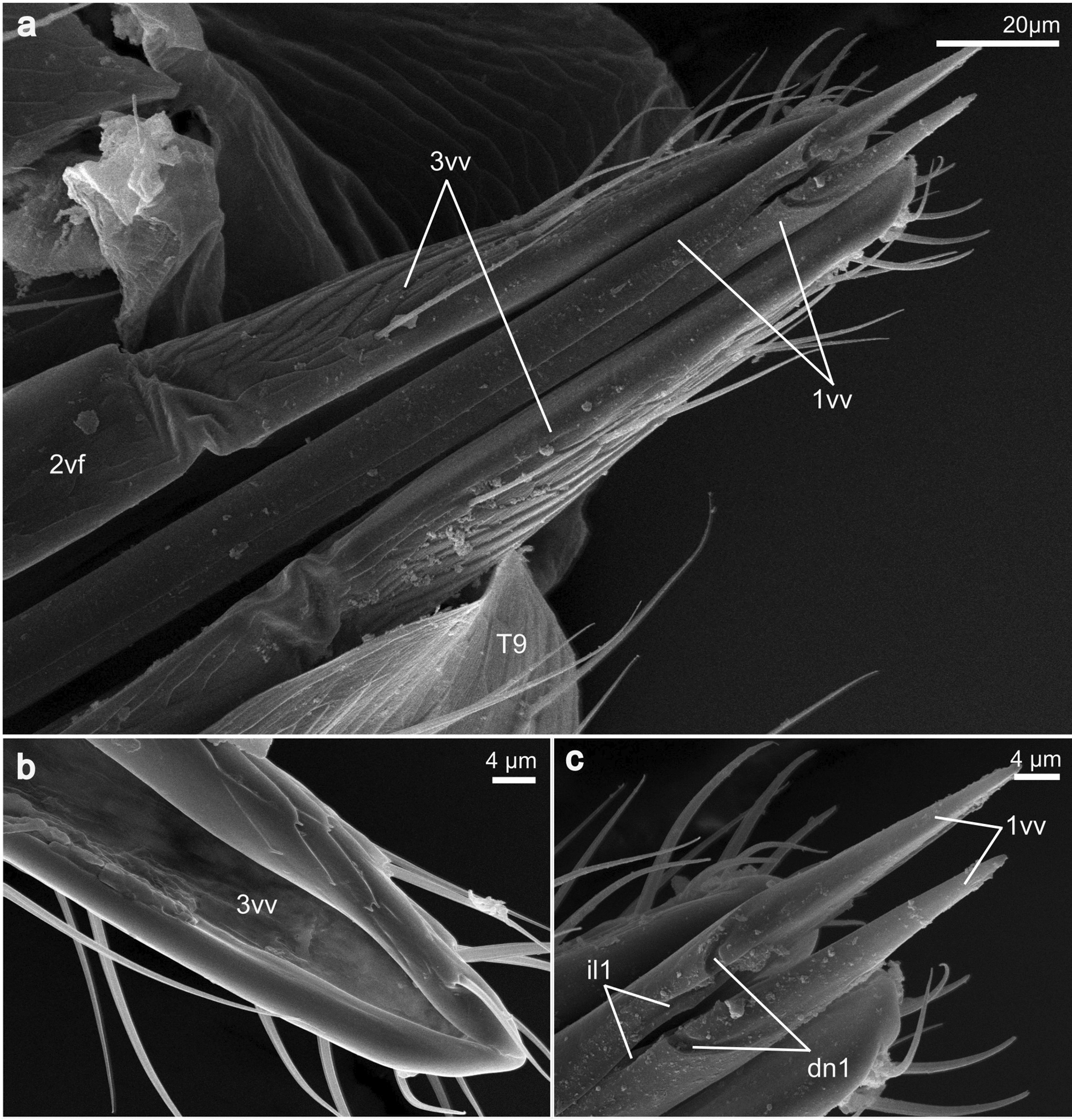
SEM images of the posterior part of the ovipositor of *Microterys flavus*, i.e. the 3rd valvulae and the apex of the terebra. **a** Terebra apex and the laterally sheathing 3rd valvulae (terebra slightly extended, ventral view). **b** View onto the interior surface of the 3rd valvulae (from medial). **c** Detailed view of the apex of the 1st valvulae with the distal notch and the interlock of the 1st valvulae (ventral view). *Abbreviations*: 1vv: 1st valvulae; 2vf: 2nd valvifer; 3vv: 3rd valvulae; dn1: Distal notch of the 1st valvula; il1: Interlock of the 1st valvulae; T9: Female T9

**Fig. 5:**
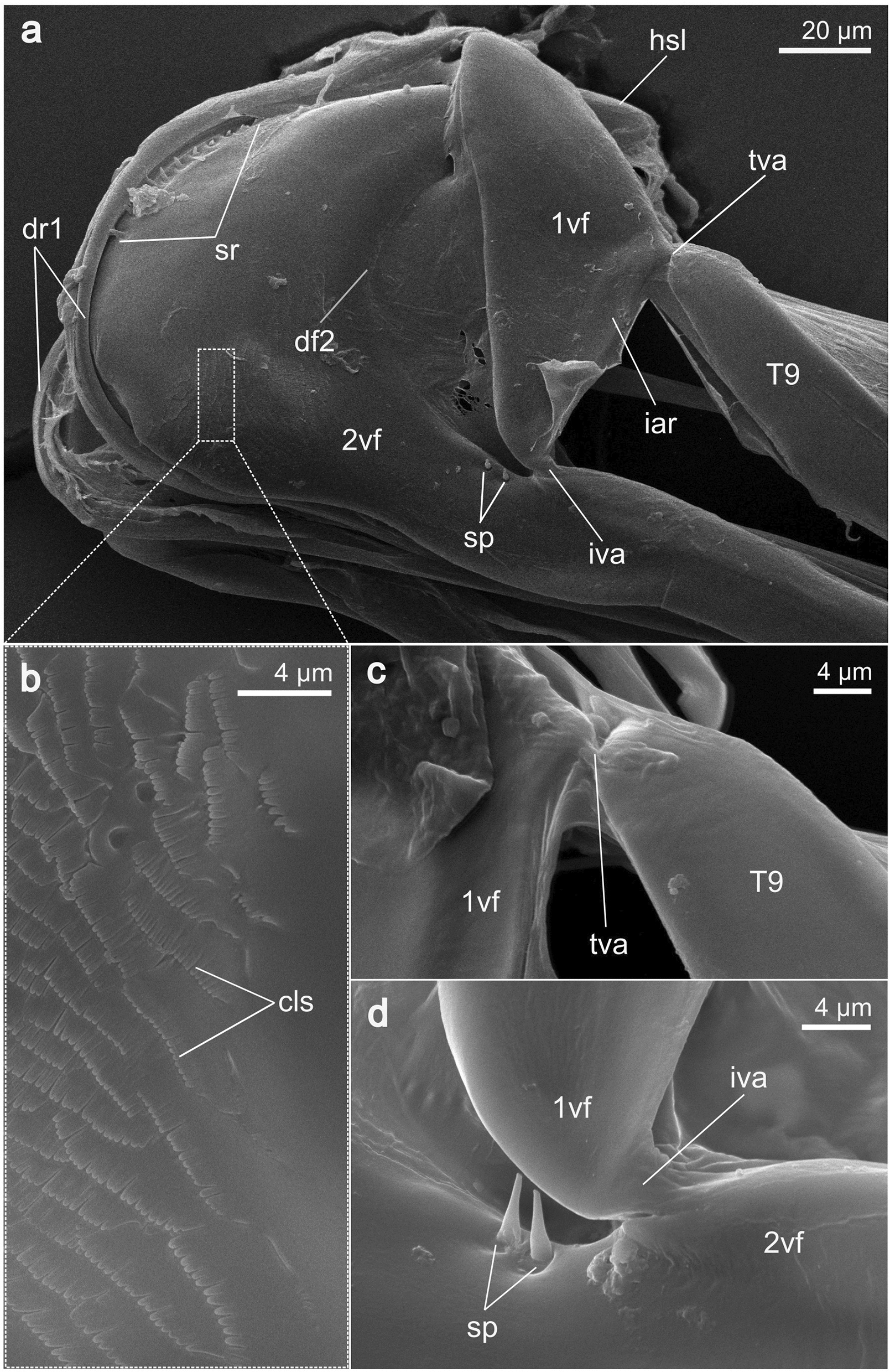
SEM images of the various ovipositor elements of *Microterys flavus*. **a** Anterior part of the ovipositor, showing the 1st valvifer and its articulation to the female T9 and 2nd valvifer (lateral view). The 1st valvifer is continuous with the 1st valvulae through the dorsal ramus. **b** Detailed view of the anteroventrally situated field of comb-shaped scales. **c** Detailed view of the tergo-valvifer articulation, connecting the 1st valvifer and the female T9. **d** Detailed view of the intervalvifer articulation, connecting the 1st valvifer and 2nd valvifer. The sensillar patch lies adjacent to the articulation. *Abbreviations*: 1vf: 1st valvifer; 2vf: 2nd valvifer; cls: Comb-like scales; df2: Dorsal flange of 2nd valvifer; hsl: Hook-shaped lobe of 2nd valvifer; dr1: Dorsal ramus of the 1st valvula; iar: Interarticular ridge of the 1st valvifer; iva: Intervalvifer articulation; sr: Sensillar row of the 2nd valvifer; sp: Sensillar patch of the 2nd valvifer; T9: Female T9; tva: Tergo-valvifer articulation

**Fig. 6:**
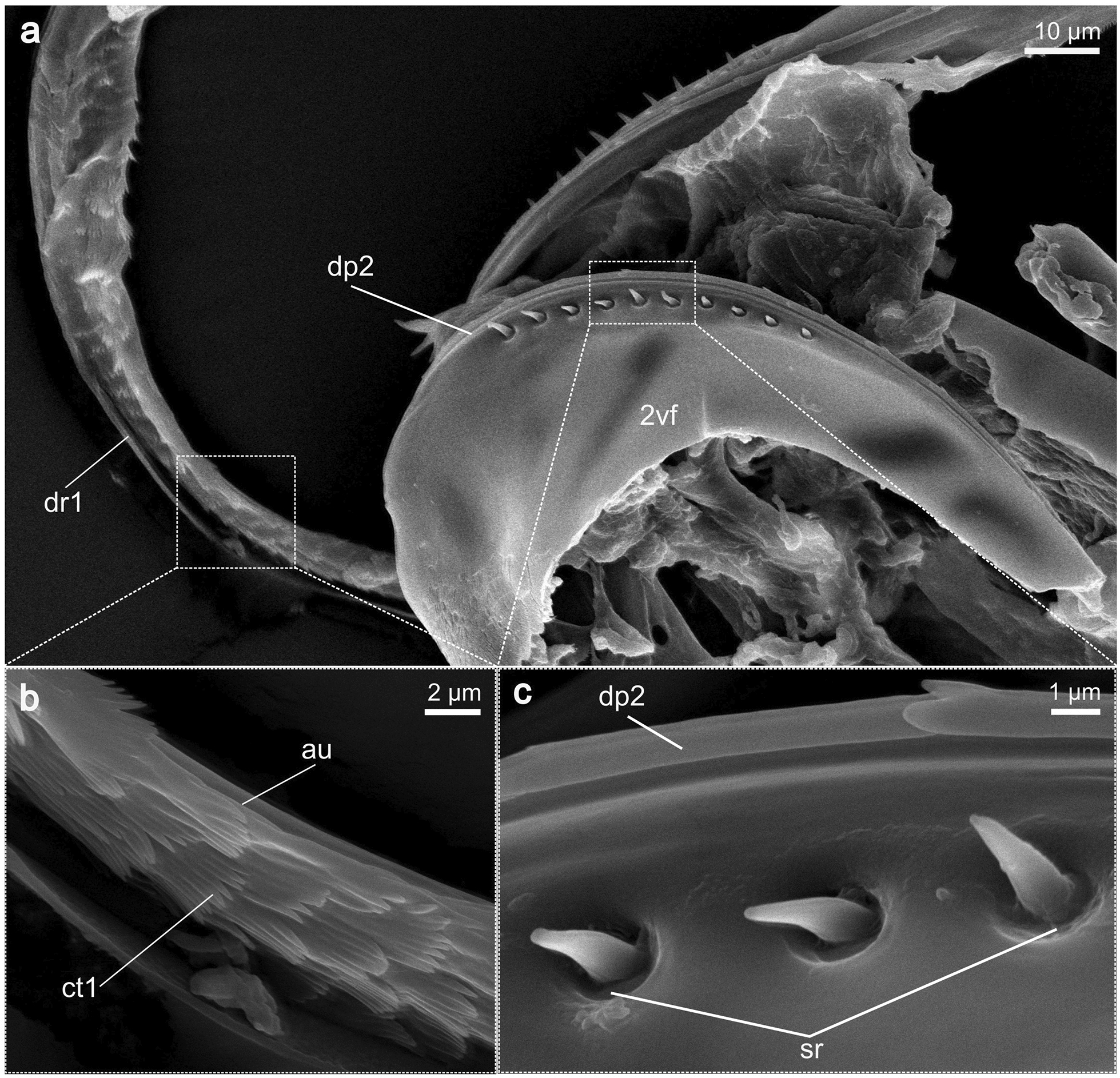
SEM images of the anterodorsal region of the 2nd valvifer of *Microterys flavus*. During preparation, the dorsal ramus of the 1st valvifer was detached from its original position (around the anterodorsal margin of the 2nd valvifer) to reveal the dorsal projection of the 2nd valvifer. **a** 2nd valvifer, showing a row of sensilla along the dorsal projection (dorsolateral view). **b** Detailed view of the ventral structure of the dorsal ramus, showing the distally directed ctenidia. **c** Detailed view of the dorsal projection of 2nd valvifer, which resembles the elongation of the rhachis of the 2nd valvula. A part of the row of sensilla is visible below. *Abbreviations*: 2vf: 2nd valvifer; au: Aulax; ct1: Ctenidia-like structures at dorsal ramus; dp2: Dorsal projection of 2nd valvifer; dr1: Dorsal ramus of the 1st valvula; hsl: Hook-shaped lobe of 2nd valvifer; rh: Rhachis; sr: Sensillar row of the 2nd valvifer

**1st valvula** (1vv; Figs. 3, 4a, c): The 1st valvula is basally continuous with the corresponding 1st valvifer via their dorsal ramus (dr1; Figs. 5a, 6a). Together, the paired 1st valvulae form the ventral part of the terebra. At the very base of each of the 1st valvulae, an entrance into a canal is visible, which suggests that the 1st valvulae possess a lumen here, extending throughout its length (lu1; cf. Fig. 8d). The dorsal side of each of the 1st valvulae acts as the ventral surface of the egg canal and bears complex microsculptures, typically in the form of comb-like patterns, called the ctenidia (ct; Fig. 3d). These processes are distally oriented and cover the egg canal wall throughout its length but become less distinctly formed towards the distal end of the 1st valvula (cf. Fig. 3). The apex of the 1st valvula is scattered with multiple sensilla (se; Fig. 3a–c). Towards the apex, the medioventral margins of both 1st valvulae fold inwards (il1; Fig. 4c). In addition, the apical area of each 1st valvula carries an obliquely transverse notch on its ventral side (dn1; Figs. 3a, 4c), running towards the apex).

**2nd valvula** (2vv; Fig. 3): The 2nd valvula forms the dorsal element of the terebra. Proximally, it consists of two halves connected by a transversely striate band, termed the laminated bridge (lb; Fig. 8c) (cf. [40]). Because of the finite spatial resolution achievable for a given field-of-view required to depict the complete sample, the exact construction and cross-section of the 2nd valvula can only be described to a limited extent here. The entire proximal section of the 2nd valvula is divided into two halves that overlap asymmetrically along almost their full length (2vv; arrows in Fig. 9c). The 2nd valvula halves are fused only at their very apex (cf. Fig. 3c, d). At its proximal base, the 2nd valvula forms laterally thickened bulbs (blb; Fig. 8a, b). A paired apodeme, called the processus musculares (prm; Fig. 8c) emerges dorsally from the anteriorly directed horn-like structures of the bulbs and acts as an attachment site for the musculature. A second processus termed the processus articularis (pra; Fig. 8c, d) emerges laterally on both sides of the bulbs and acts not only as a muscle attachment site, but also as the medial part of the basal articulation, thus playing a role in articulating the 2nd valvula with the 2nd valvifer. Whereas the 1st valvulae lack any teeth-like structures, the apex of the 2nd valvula bears multiple sawteeth (st; Fig. 3a–c) on its dorsal margin. These teeth are arranged laterally and alternately along the margin and decrease in size towards the tip, where two medially situated sawteeth can be found. Unlike the 1st valvulae, no sensilla were detected at the apex of the 2nd valvula. However, this does not exclude the presence of such structures, which might not be recognisable from the given angle or are simply too small to be seen.

**Fig. 7:**
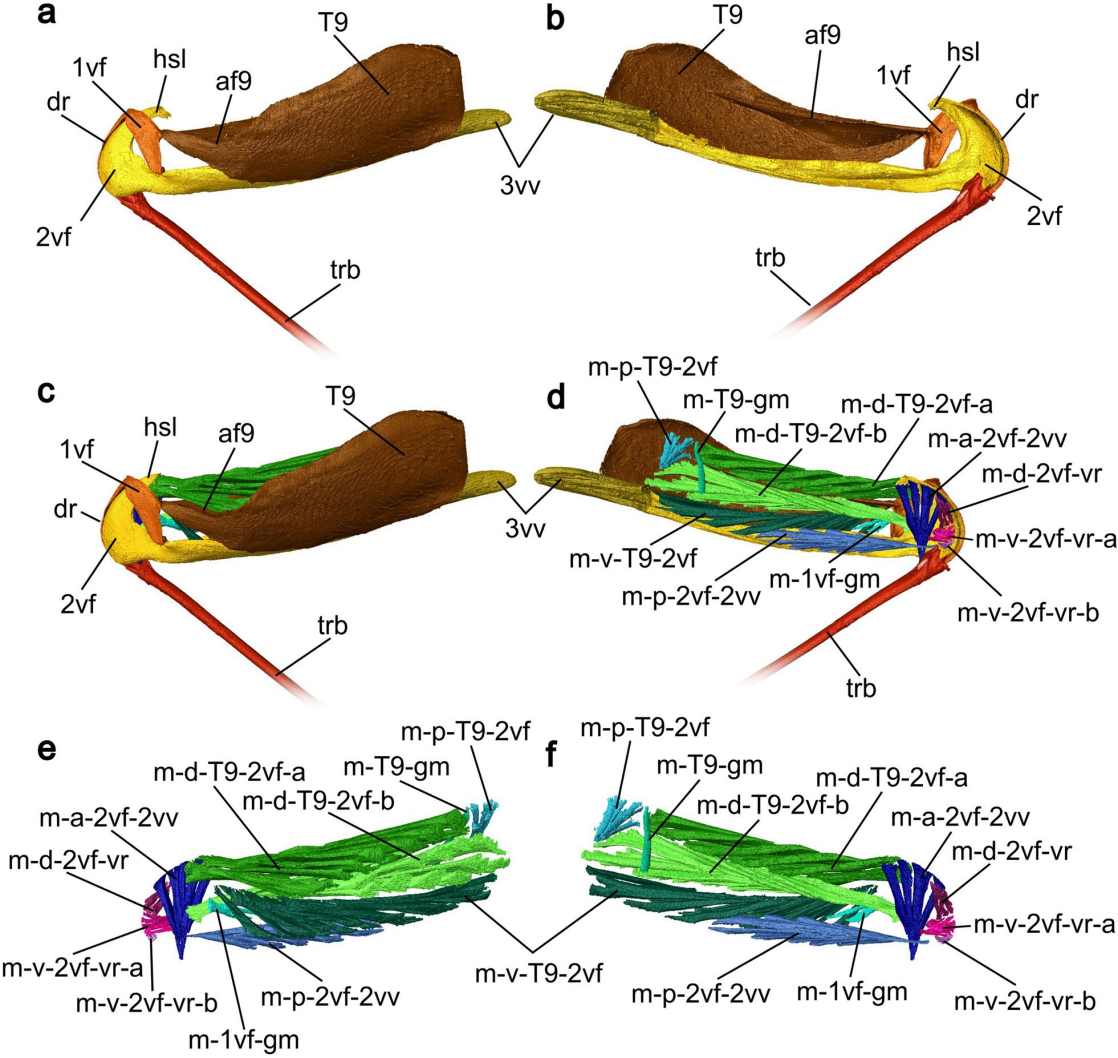
3D model of the musculoskeletal ovipositor system of *Microterys flavus* in an active probing position (terebra partly depressed) based on SR-µCT data (**a**, **c**, **e** lateral view, left is anterior; **b**, **d**, **f** medial view, left is posterior). The model considers only the left half of the ovipositor system and can be mirrored along the longitudinal axis. **a, b** Cuticular elements of the ovipositor system. **c, d** Musculoskeletal ovipositor system, including all inherent cuticular elements and musculature. **e, f** Ovipositor muscles actuating the ovipositor system. *Abbreviations*: 1vf: 1st valvifer; 2vf: 2nd valvifer; 3vv: 3rd valvula; af9: Anterior flange of the female T9; dr1: Dorsal ramus of the 1st valvula; hsl: Hook shaped lobe of 2nd valvifer; m-1vf-gm: 1st valvifer-genital membrane muscle; m-a-2vf-2vv: Anterior 2nd valvifer-2nd valvula muscle; m-d-2vf-vr: Dorsal 2nd valvifer-venom gland reservoir muscle; m-d-T9-2vf-a: Dorsal T9-2nd valvifer muscle (part a); m-d-T9-2vf-b: Dorsal T9-2nd valvifer muscle (part b); m-p-2vf-2vv: Posterior 2nd valvifer-2nd valvula muscle; m-p-T9-2vf: Posterior T9-2nd valvifer muscle; m-T9-gm: T9-genital membrane muscle; m-v-2vf-vr-a: Ventral 2nd valvifer-venom gland reservoir muscle (part a); m-v-2vf-vr-b: Ventral 2nd valvifer-venom gland reservoir muscle (part b); m-v-T9-2vf: Ventral T9-2nd valvifer muscle; T9: Female T9; trb: Terebra

**Fig. 8:**
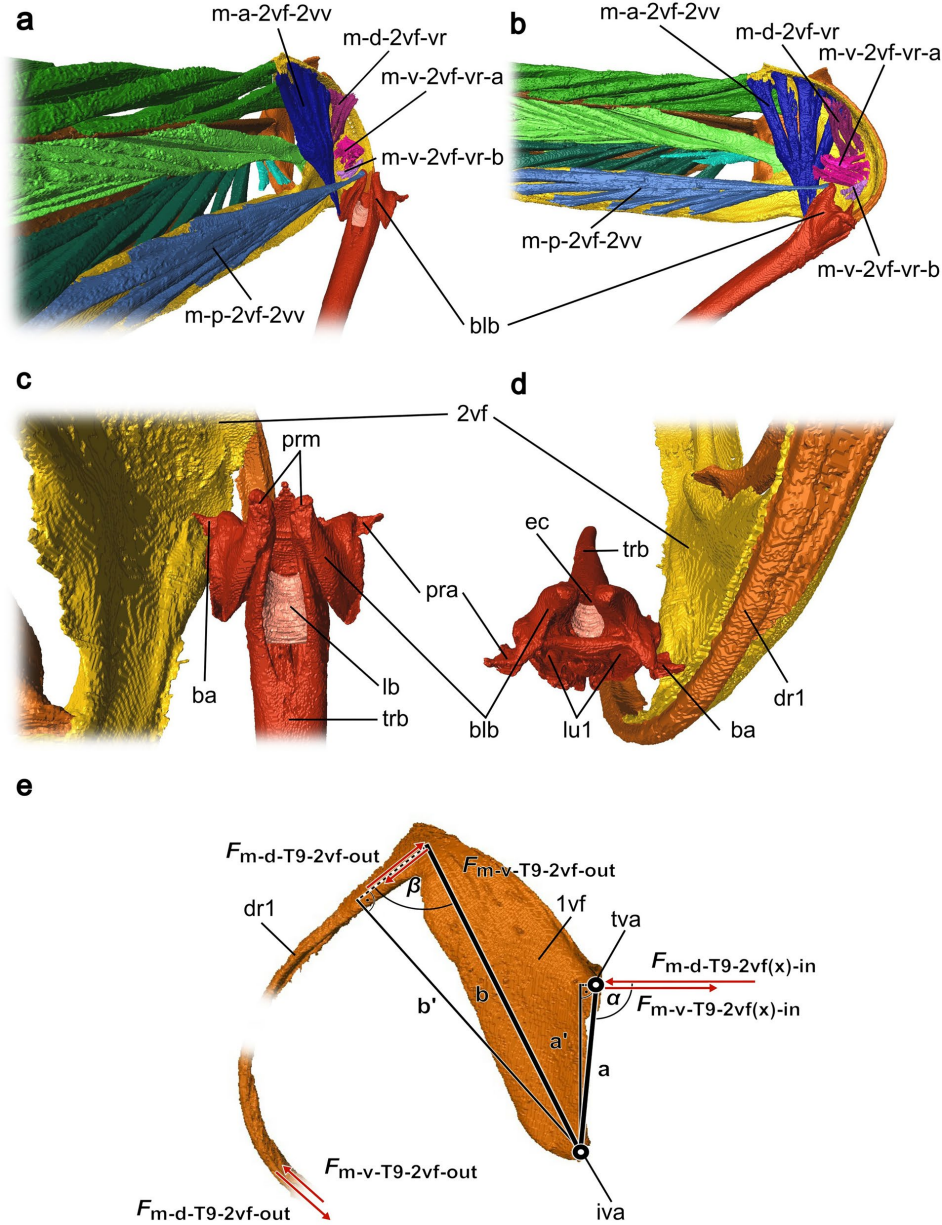
3D model based on SR-μCT data with detailed images of various anterior elements of the ovipositor system of *Microterys flavus* in an active probing position (terebra partly depressed). The model considers only the left half of the ovipositor system and can be mirrored along the longitudinal axis. **a** Muscles inserting at the bulbs of the 2nd valvula (posteromedial view). Depression and retraction of the terebra is actuated by the posterior 2nd valvifer-2nd valvula muscle and the anterior 2nd valvifer-2nd valvula muscle. **b** Muscles inserting at the bulbs of the 2nd valvula (medial view). **c** Dorsal view on the base of the terebra, featuring the bulbs, the laminated bridge and muscle attachment sites; the processus articularis is the insertion site for the anterior 2nd valvifer-2nd valvula muscle, whereas the processus musculares is the insertion site of the posterior 2nd valvifer-2nd valvula muscle. **d** Base of the terebra from anterior, featuring the bulbs, the insertion sites for the anterior and posterior 2nd valvifer-2nd valvulae muscle, and the entrance into the egg canal and the lumen of the 1st valvulae. **e** Lateral view of the 1st valvifer (left is anterior) including the dorsal ramus of the 1st valvula. Acting muscle forces are visualized by solid red arrows. Under the simplified assumption that the 2nd valvifer and the female T9 are guided and cannot twist but only telescopically slide towards or against each other along the anterior–posterior axis, the input force vectors *F*_m-d-T9-2vf(x)-in_ and *F*_m-v-T9-2vf(x)-in_ act in the same plane only at the tergo-valvifer articulation. The anatomical inlever a is the distance between the tergo-valvifer articulation (where the force is applied) and the intervalvifer articulation (pivot point); the effective (= mechanical) inlever, is a’. The anatomical outlever b is the distance between the intervalvifer articulation and the point at which the 1st valvifer continues as dorsal ramus of the 1st valvula; the effective outlever is b’. The 1st valvifer acts as a lever transferring the resulting pro- or retraction forces, *F*_m-d-__T9__-2vf-out_ and *F*_m-v-T9-2vf-out_, to the dorsal ramus of the 1st valvula. *Abbreviations*: 1vf: 1st valvifer; 2vf: 2nd valvifer; ba: Basal articulation; blb: Bulbs; dr1: Dorsal ramus of the 1st valvula; ec: Egg canal; *F*: force; *F*_(x)_: horizontal vector component of a force; iva: Intervalvifer articulation; lb: Laminated bridge; lu1: Lumen of 1st valvulae; m-a-2vf-2vv: Anterior 2nd valvifer-2nd valvula muscle; m-d-2vf-vr: Dorsal 2nd valvifer-venom gland reservoir muscle; m-p-2vf-2vv: Posterior 2nd valvifer-2nd valvula muscle; m-v-2vf-vr-a: Ventral 2nd valvifer-venom gland reservoir muscle (part a); mv-2vf-vr-b: Ventral 2nd valvifer-venom gland reservoir muscle (part b); pra: Processus articularis; prm: Processus musculares; tva: Tergo-valvifer articulation; trb: Terebra

**Fig. 9:**
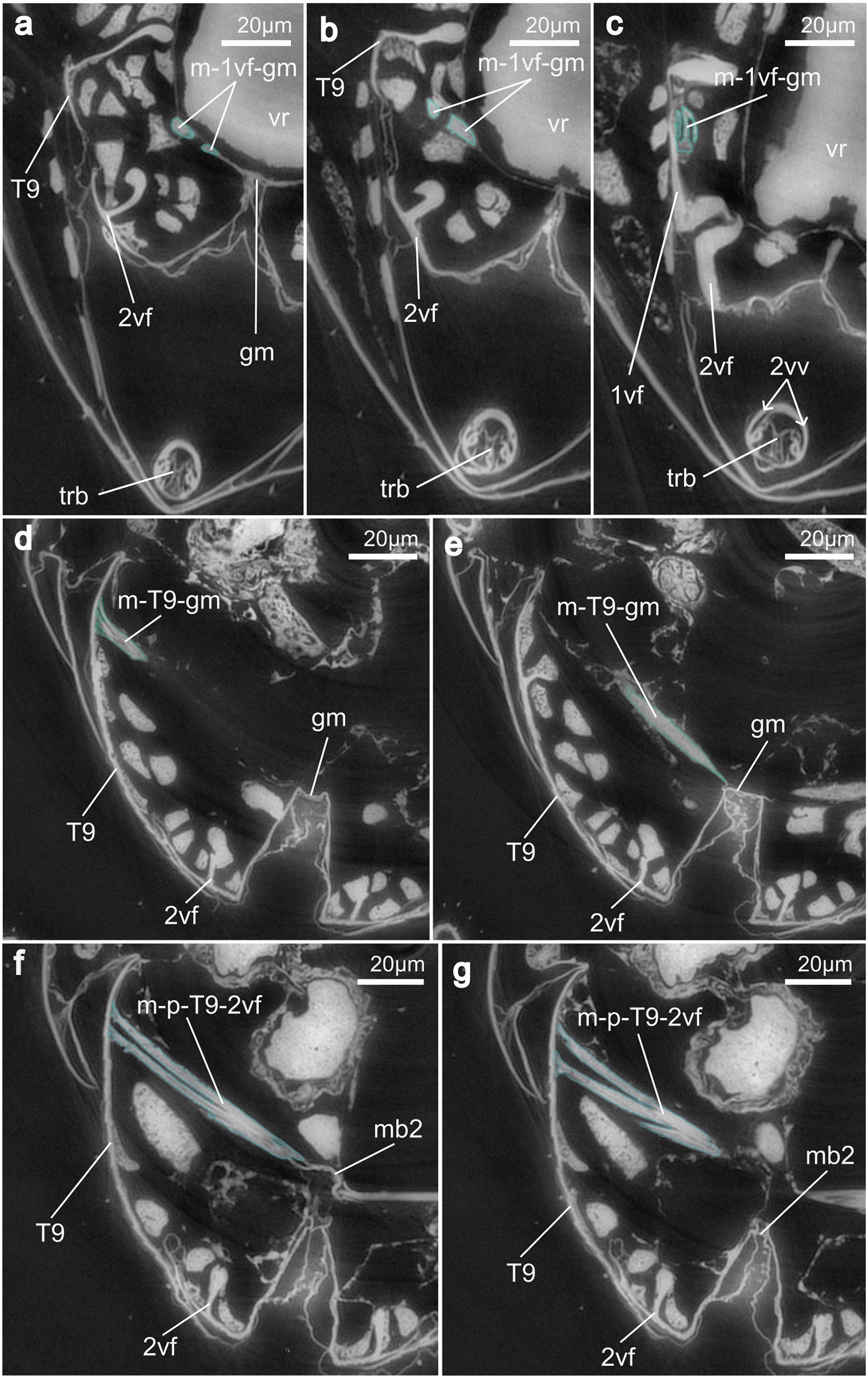
SR-μCT images of virtual transversal slices through one half of the ovipositor and the anteroventral metasoma of *Microterys flavus*. This plate illustrates the attachment areas of the 1st valvifer-genital membrane muscle, the T9-genital membrane muscle, and the posterior T9–2nd valvifer muscle. **a–c** Muscle m-1vf-gm emerges from the 1st valvifer (at interarticular ridge) and inserts at the genital membrane (from a to c: posterior to anterior). **d, e** Muscle m-T9-gm emerges from the female T9 and inserts at the genital membrane (from d to e: posterior to anterior). **f, g** Muscle m-p-T9-2vf emerges from the female T9 and inserts at the median bridge, posterodorsally connecting both ends of the 2nd valvifer (from f to g: posterior to anterior). *Abbreviations*: 1vf: 1st valvifer; 2vf: 2nd valvifer; 2vv: 2nd valvula; gm: Genital membrane; m-1vf-gm: 1st valvifer-genital membrane muscle; m-p-T9-2vf: Posterior T9–2nd valvifer muscle; m-T9-gm: T9-genital membrane muscle; mb2: Median bridge; T9: Female T9; trb: Terebra; vr: Venom gland reservoir of the 2nd valvifer

**Terebra** (trb; Figs. 2c–e, g, 7a–d, 9a–c)**:** The paired 1st valvulae make up the ventral part of the terebra, whereas the 2nd valvula forms its dorsal element. The 1st valvulae are each interlocked with the 2nd valvula through a longitudinal interlocking mechanism called the olistheter. The aulax (au; Figs. 3b–d), i.e. the groove that runs sublaterally along the dorsal margin of the 1st valvulae, therefore holds the corresponding tongue, called the rhachis (rh; Fig. 3b), which is carried ventrally on both sides of the 2nd valvula. Together, they form the egg canal (ec; Fig. 8d), which has its origin in the bulbous region (cf. Fig. 8a–b) and runs through the terebra. The terebra maintains a constant width across almost its full length but tapers acicularly at its apex.

**3rd valvula** (3vv; Figs. 2b–d, 4a, b, 7a–d): The 3rd valvula is attached to the posterior end of the 2nd valvifer and is connected to it via a flexible membrane (cf. Figs. 4a, 7b). The relatively short semi-tubular 3rd valvula reaches only slightly beyond the posterior edge of the female T9 (cf. Fig. 7a–d). The exterior surface of the 3rd valvula is covered entirely by scales pointing distally. Furthermore, multiple distally pointing sensilla are scattered along the surface of the 3rd valvula and tend to concentrate towards the apex (cf. Fig. 4). The medial surface is smooth and seems to lack any sensory organs (cf. Fig. 4b). Being sheaths, they are arranged laterally around the terebra on each side when retracted and fully envelope its apex.

**1st valvifer** (1vf; Figs. 5a, c, d, 7a–c, 9c): Viewed laterally, the 1st valvifer resembles an elongated triangle with rounded edges. Starting from the anterodorsal angle, it extends into the dorsal ramus of the 1st valvula (dr1; Figs. 5a, 6a, 7a–c, 8d), which runs around the anterodorsal margin of the 2nd valvifer. Via the dorsal ramus, the 1st valvifer is continuous with their respective 1st valvula. The dorsal ramus also bears an aulax (au; Fig. 6b) that is interlocked with the rhachis-like dorsal projection of the 2nd valvifer (dp2; Fig. 6a, c), thus forming an extension of the olistheter. Along the dorsal ramus, the interior surface is covered with fine leaf-like scales (ct1; Fig. 6b) with a shape resembling that of the ctenidia situated along the medial walls of the egg canal (ct; Fig. 3d). The 1st valvifer lies between the 2nd valvifer and the female T9 and is hinged to both elements via two articulations, i.e. the intervalvifer and the tergo-valvifer articulation, respectively. Between the two articulations, the 1st valvifer is strengthened, forming the interarticular ridge (iar; Fig. 5a).

**Fig. 10:**
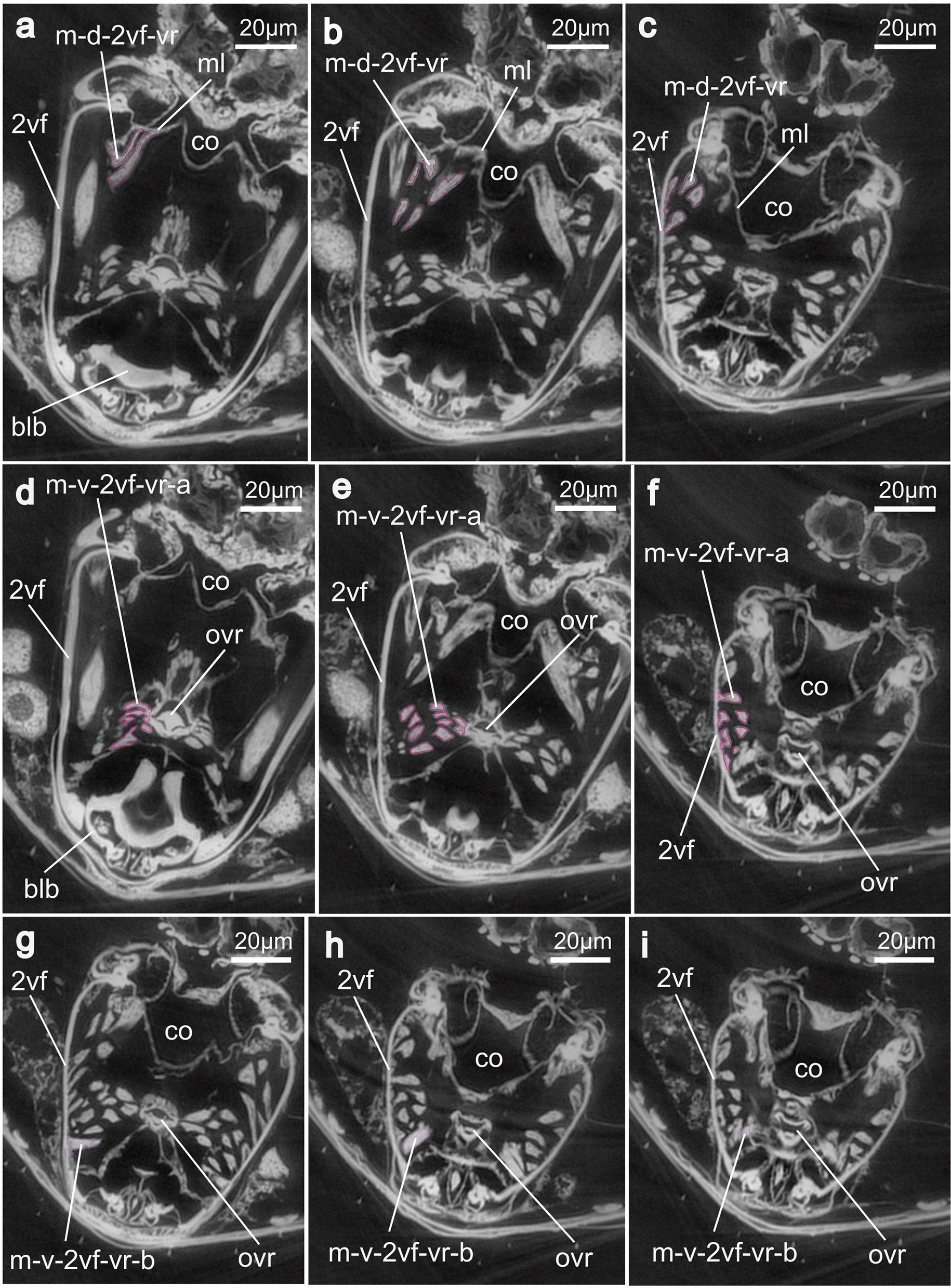
SR-μCT images of virtual transversal slices, depicting a section of the metasoma of *Microterys flavus* including the full ovipositor. This plate illustrates the attachment areas of the dorsal 2nd valvifer-venom gland reservoir muscle and the ventral 2nd valvifer-venom gland reservoir muscle (parts a, b). **a–c** m-d-2vf-vr emerges anteromedially from the 2nd valvifer and inserts at a membranous layer above the bulbs of the terebra (from a to c: posterior to anterior). **d–f** mv-2vf-vr-a emerges from the 2nd valvifer (ventrally to m-2vf-vr) and inserts laterally at the orifice of the venom gland reservoir (from d to f: posterior to anterior). **g–i** m-v-2vf-vr-b emerges from the 2nd valvifer (ventrally to m-d-2vf-co) and inserts laterally at the orifice of the venom gland reservoir, ventrally to the m-v-2vf-vr-a (from g to i: posterior to anterior). *Abbreviations*: 2vf: 2nd valvifer; blb: Bulbs; m-d-2vf-vr: Dorsal 2nd valvifer-venom gland reservoir muscle; m-v-2vf-vr-a: Ventral 2nd valvifer-venom gland reservoir muscle (part a); m-v-2vf-vr-b: Ventral 2nd valvifer-venom gland reservoir muscle (part b); ml: Membranous layer; ovr: Orifice of the venom gland reservoir

**2nd valvifer** (2vf; Figs. 4a, 5a, d, 6a, 7a–c, 8c–d, 9a–g, 10a–i): The 2nd valvifer can be roughly divided into two areas according to their shape. Its anterior part has a semi-circular curved form that opens, in the posterior direction, into a hook-shaped lobe (hsl; Figs. 5a, 7b–c) pointing posteriorly. The posterior part of the 2nd valvifer is elongated and runs medially underneath the female T9 and makes up almost a third of the length of the metasoma (cf. Fig 7a–b). The anterodorsal margin of the 2nd valvifer bears the dorsal projection of the 2nd valvifer (dp2; Fig. 6a, c). This element represents an extension of the rhachis of the 2nd valvula (rh; Fig. 3b) and is interlocked with the aulax of the dorsal ramus via an olistheter-like interlocking system. Laterally to the dorsal projection, the 2nd valvifer bears an arcuate row of fine sensilla (sr; Fig. 5a, 6c), running right beneath the dorsal ramus of the 1st valvula (dr1; Figs. 5a, 6a, 7a–c, 8d). On the exterior surface of the anteroventral end of the 2nd valvifer, it additionally carries a field of comb-shaped scales (cls; Fig. 5b) that point posterodorsally. At its most posterior end, the 2nd valvifer bears a 3rd valvula (3vv; Figs. 2b–d, 4a–b, 7a–d). The transition between the two structures seems to be continuous and is clearly distinguishable when investigated by SEM (cf. Fig. 4a). Both 2nd valvifers are medially connected by a conjunctiva that is named the genital membrane (gm; Fig. 9a, d, e) and that is attached to the ventral margin of both the valvifers and arches above the 2nd valvula (in resting position). Moreover, the 2nd valvifers are also connected by the median bridge (mb2; Fig. 9f–g) emerging from the posterodorsal ends of both 2nd valvifers.

**Female T9** (T9; Figs. 4a, 5a, c, 7a–c, 9a, b, d–g): The female T9 is homologous to the last abdominal tergite and is elongated anteriorly into the metasoma. It is relatively wide for most of its posterior area, where it has a rectangular form, but becomes narrower anteriorly, where it points in an anterodorsal direction. The cordate apodeme emerges medially at the anterior end of the female T9 and is mainly recognisable as a local bulge-like structure. Starting dorsally at the anterior end of the female T9, a significant medial ridge is formed, running medially along almost its full length. The dorsal margin of the female T9 bears the anterior flange (af9; Fig. 7a–c). The exterior side of the female T9 shows a rough surface that carries several hairs at the posterior end.

#### Articulations between cuticular elements of the ovipositor

**Basal articulation**, connecting the 2nd valvula with the 2nd valvifer (ba; Fig. 8c–d): This rotational articulation connects the ball-like processus articularis laterally on the bulb (blb; Figs. 8a–b, 10a, d) of the 2nd valvula to the socket-like pars articularis of the anteroventral part of the 2nd valvifer. This articulation allows a rotational movement (depression and elevation) of the 2nd valvula and thereby of the entire terebra.

**Intervalvifer articulation**, connecting the 1st valvifer with the 2nd valvifer (iva; Fig. 5a, d): The intervalvifer articulation connects the ventral corner of the 1st valvifer to the 2nd valvifer. Together with the ventrally situated tergo-valvifer articulation, it enables a back-and-forth rotation of the 1st valvifer along the sagittal plane. Adjacent to the intervalvifer articulation lies a sensillar patch comprising a pair of sensilla (sp; Fig. 5a, d).

**Tergo-valvifer articulation**, connecting the 1st valvifer with the female T9 (tva; Fig. 5a, c): The tergo-valvifer articulation connects the posterodorsal corner of the 1st valvifer with the most anterior protrusion of the female T9 near the cordate apodeme. Together with the intervalvifer articulation, it allows the previously mentioned rotational movement of the 1st valvifer.

#### Ovipositor muscles

The ovipositor system of *M. flavus* comprises a set of nine paired muscles, whereby two muscles (i.e. the dorsal T9-2nd valvifer muscle and the ventral 2nd valvifer-venom gland reservoir muscle) each consist of two bundles designated as parts a and b (m-d-T9-2vf-a/b, m-v-2vf-vr-a/b; Figs. 7d–f, 8a, b). Three of these ovipositor muscles, namely the 1st valvifer-genital membrane muscle (m-1vf-gm), the ventral 2nd valvifer-venom gland reservoir muscle (m-v-2vf-vr-a/b), and the T9-genital membrane muscle (m-T9-gm), were only recently discovered for Chalcidoidea [32] and are described here for the first time in an encyrtid wasp.

**1st valvifer-genital membrane muscle** (m-1vf-gm; Fig. 7d–f, 9a–c): The small 1st valvifer-genital membrane muscle is the only ovipositor muscle that attaches to the 1st valvifer. It originates medially at the posteroventral margin of the 1st valvifer, right between the intervalvifer and the tergo-valvifer articulations (iva/tva; Fig. 5a, c, d), where it attaches close to the interarticular ridge (iar; Fig. 5a) and runs medially towards the genital membrane (see Fig. 9a–c). This muscle is described here for the first time for a member of Encyrtidae.

**Dorsal 2nd valvifer-venom gland reservoir muscle** (m-d-2vf-vr; Figs. 7d–f, 8a–b, 10a–c): The dorsal 2nd valvifer-venom gland reservoir muscle originates at the medial surface of the anterodorsal end of the 2nd valvifer. From here, it has a dorsal orientation, whilst running into the medial plane of the ovipositor where it attaches to a membranous layer (ml; Fig. 10a–c) that is located at the anterodorsal surface of the venom gland reservoir and that separates the reservoir from the dorsally situated common oviduct (co; Fig. 10). This layer lies dorsally above the bulbous region of the terebra (see Figs. 8a–b, 10a). This muscle has previously been described in various pteromalids [32, 41] but is, to the best of our knowledge, newly described here for Encyrtidae.

**Ventral 2nd valvifer-venom gland reservoir muscle part a/b** (m-v-2vf-vr-a/b; Figs. 7d–f, 8a–b, 10d–i): The small ventral 2nd valvifer-venom gland reservoir muscle comprises of two distinct bundles that originate in direct proximity of each other. Part a (m-v-2vf-vr-a; Figs. 7d–f, 8a–b, 10d–f) originates ventrally to m-d-2vf-vr at the medial surface of the anteroventral end of the 2nd valvifer. It runs in a straight plane towards the centre of the ovipositor where it inserts laterally at the orifice of the venom gland reservoir (ovr; Fig. 10d–i). Part b (m-v-2vf-vr-b; Figs. 7d–f, 8a–b, 10g–i) inserts at the medial surface of the anteroventral end of the 2nd valvifer, adjacent to the basal articulation. Like the m-v-2vf-vr-a, this bundle is medially directed but shows a slightly anterior orientation. It also inserts laterally at the orifice of the venom gland reservoir in the direct vicinity of the bulbs of the terebra and before the orifice of the venom gland reservoir enters the common oviduct (cf. Figs. 8a–b, 10a–i). The muscle is thinner and smaller than the two described above. Both muscles, i.e. m-d-2vf-vr and m-v-2vf-vr, have a short and stout form and do not fan out but keep a constant width. To the best of our knowledge, these two muscles have not yet been reported in any encyrtid wasp hitherto and have only been recently described for a member of Chalcidoidea, namely the pteromalid *Lariophagus distinguendus* (Förster, 1841) [32]. A similar set of muscles has been described in ants [42–44].

**Anterior 2nd valvifer-2nd valvula muscle** (m-a-2vf-2vv; Figs. 7d–f, 8a–b): The anterior 2nd valvifer-2nd valvula muscle originates at the medial surface of the anterodorsal arch of the 2nd valvifer, where it is attached to the dorsal flange (df2; Fig. 5a). From here, it runs in a ventral direction to the processus articularis (pra; Fig. 8c–d), located laterally to the proximal bulbous end of the 2nd valvula. The muscle fans out strongly along its ventral–dorsal axis (see Fig. 8b).

**Posterior 2nd valvifer-2nd valvula muscle** (m-p-2vf-2vv; Figs. 7d–f, 8a–b): The posterior 2nd valvifer-2nd valvula muscle originates at the medial surface along the ventral part of the 2nd valvifer, at the area between the anterior and posterior end, and inserts at the processus musculares (prm; Fig. 8c), i.e. the anterodorsally directed processus of the bulbs of the 2nd valvula, putatively through a sclerotized tendon (see Fig. 8a–b).

**Dorsal T9-2nd valvifer muscle part a/b** (m-d-T9-2vf-a/b; Fig. 7d–f): The dorsal T9-2nd valvifer muscle forms two distinct bundles that have similar proportions and that differ in their areas of origin and insertion. One part (part a; m-d-T9-2vf-a; Fig. 7d–f) originates along both the dorsolateral and dorsomedial surface of the female T9 along its medial ridge and inserts at the hook-shaped lobe of the 2nd valvifer (hsl: Figs. 5a, 7a–c) along its dorsal flange. The second part (part b; m-d-T9-2vf-b; Fig. 7d–f) lies ventrally to the first part and originates at the medial surface of the female T9, ventrally to the medial ridge of the T9. It is bundled into a narrow strand before inserting at the anterior section of the dorsal flange of the 2nd valvifer, located ventrally to the insertion region of part a. Combined, both parts form the largest muscle in the ovipositor system of *M. flavus*.

**Ventral T9-2nd valvifer muscle** (m-v-T9-2vf; Fig. 7d–f): The ventral T9-2nd valvifer muscle arises at the cordate apodeme, situated around the anterior end of the female T9. The muscle largely extends in width and inserts along almost the full posterior part of the dorsal flange of the 2nd valvifer (df2; Fig. 5a).

**T9-genital membrane muscle** (m-T9-gm; Figs. 7d–f, 9d, e): The thinnest muscle of the ovipositor system is the T9-genital membrane muscle. Here, we describe this muscle for the first time for Encyrtidae. The muscle originates at the medial surface of the posterodorsal part of the female T9 and inserts at the genital membrane between the 2nd valvifers (gm; Fig. 9a, d, e), i.e. the structure that connects the ventral margins of both 2nd valvifers.

**Posterior T9-2nd valvifer muscle** (m-p-T9-2vf; Figs. 7d–f, 9f, g): The short and thin posterior T9-2nd valvifer muscle arises from the posterodorsal region of the female T9, anterior to the origin of the m-T9-gm. It then runs medially to insert at the median bridge (mb2; Fig. 9f, g), i.e. the structure that connects both the posterodorsal ends of the 2nd valvifers.

## Discussion

We have combined behavioural observations and morphological investigations to thoroughly understand the musculoskeletal ovipositor system of the parasitoid encyrtid wasp *Microterys flavus*. Our intention is to improve our knowledge of the ecomorphological importance of this system, including its functional morphology in interaction with its scale insect host. Our study is based on earlier studies on the ovipositor system of parasitoid wasps; cf. studies on the ovipositor of species belonging to Chalcidoidea: Agaonidae [25], Aphelenidae [17], Chalcididea [21, 26], Eulophidae [19], Eurytomidae [20, 27], Pteromalidae [22, 24, 32], Torymidae [18, 28], Ceraphronoidea [45]; Cynipoidea: Cynipidae [46], Figitidae [47]; Ichneumonoidea: Braconidae [48, 49], Ichneumonidae [50] (also see [5–7, 10]), and in particular on the studies focusing on the mechanics and mode of function of the ovipositor (cf. [32, 47, 48]). Eggs et al. [32] provided the first thorough investigation of the mode of function of the musculoskeletal ovipositor system of a chalcidoid wasp, the pteromalid *L. distinguendus* in particular, and also highlighted the mechanisms of terebra steering movements. The present study on *M. flavus*, however, is the first examination of the mode and function of the musculoskeletal ovipositor system in a species belonging to the Encyrtidae.

### Oviposition process and employment of the terebra

As an endoparasitoid, a major part of the parasitization process in *M. flavus* occurs within the host’s body. Therefore, some phases (regarding host assessment and envenomation) cannot be distinguished by our videography analyses. In the following, we focus on the various phases of the oviposition process and discuss this in relation to the involved morphological structures.

**Search for a potential host:** The way in which *M. flavus* locates a potential host is not yet known. However, studies on closely related encyrtid wasps have revealed that the wasps use both olfactory and gustatory stimuli for host detection and evaluation [51, 52]. In the case of host-specific parasitoids, the excretions (e.g. honeydew) of the preferred host presumably serve as a directional cue for host finding [51]. Indeed, in some cases, not only the odour of the host organism itself is used for host detection, but also that of the host plant that is potentially infested by the host [52]. Once the female *M. flavus* has encountered its host, it starts to assess its suitability. Since the preferred scale insect host *Coccus hesperidum* is not concealed by any structure, the wasp is able to freely access and potentially to test its overall suitability during the first moments of contact between the wasp and the host. Therefore, it starts to skim across the dorsal shield of the host, performing "antennal drumming" (Fig. 2a; Additional file 1, min. 0:13– 0:26; cf. [34, 51, 53, 54]). We assume that the tips of the antennae are equipped with sensory organs suitable for receiving chemical stimuli from the host, as shown in a study of the encyrtid *Cheiloneurus noxius* Compere, 1925 [55].

**Penetration of the host’s skin:** Once the female *M. flavus* has detected a suitable spot on the dorsum of its host for penetration, it uses its terebra to penetrate it. A fundamental problem of such thin and needle-like structures occurs when an axial load is applied, because the terebra is about to buckle [56, 57]. This problem arises prior to drilling, as the wasp needs to push the terebra against the tough dorsal shield to fixate the apices of the 1st valvulae into the host’s dorsal shield by using their sawteeth. However, the female *M. flavus* overcomes this challenge by stabilizing the terebra with the 3rd valvulae. Moreover, the terebra even remains within the metasoma and the paired 3rd valvulae, while being brought into the drilling position (Additional file 1, min. 0:26–0:32). This is mainly achieved by bending the full metasoma downwards (see Fig. 2b) prior to the penetration process. Once the tip of the terebra is pushed and fixed at the drilling site, the metasoma is lifted again, releasing the terebra between the 3rd valvulae. The described behaviour has previously been observed in many other representatives of Chalcidoidea (cf. [18, 20, 21, 32, 40]) and hints at an additional sensory function of the 3rd valvulae. This is supported by the numerous small setae that cover its distal end, guiding the terebra to the actual drilling site. Although the length and thereby the possible function of the 3rd valvulae differ between species (cf. [17]), the sensory function is a shared trait of the 3rd valvulae amongst chalcidoid wasps. The working mechanism of both the involved muscles is discussed in more depth below (see paragraph ‘Depression and elevation of the terebra/bending of the terebra’ below). Once the terebra has been anchored at the desired drilling site, the wasp initiates the drilling process. Various previous studies on parasitoid wasps have dealt with the principles of terebra drilling [32, 48, 56–59], and a ‘push–pull’ behaviour has been observed (cf. [57–59]) being conducted by the terebra in the form of alternate reciprocal forwards-and-backwards movements of the 1st valvulae, while drilling into the substrate. This drilling method has the benefit of minimising the axial load acting on the terebra and therefore avoiding the risk of buckling. Cerkvenik et al. [56] have worked on the braconid *Diachasmimorpha longicaudata* (Ashmead, 1905) and generally divided the observed drilling action of parasitoid wasps into two distinct techniques. They hypothesize that a drilling method under alternating valvulae movements of high amplitude is used to access a tougher, high-resistance environment. On the other hand, a method of pushing the entire ovipositor into the target with only little valvulae movements is applied when a target of lower resistance is accessed. In the case of *M. flavus*, its host *C. hesperidum* is not concealed by wood or any other substrate. However, this host possesses a similar protective mechanism in the form of a waxy covering over the dorsum, which serves as a defence against predators or parasites [60]. Based on our observations, a pushing or stabbing technique supported by a rotational movement of the terebra still seems to be the most probable method used by *M. flavus* to penetrate the host’s dorsal shield. Nevertheless, a combination with an alternating back-and-forth movement of the two 1st valvulae still cannot be excluded, since a movement of the individual valvulae has not been directly observed during our recordings.

**Assessment of the host and envenomation:** For the female to assess the suitability of the host, it needs to determine whether the host has previously been used for oviposition by a competing female of the same or a different species, a process called host discrimination [61]. Additionally, an idiobiont wasp might also have the goal of locating sensitive organs and damaging them through envenomation to paralyze the host. This is achieved by probing the internal host body and by presumable chemical perception through other sensilla (se; Fig. 3a–c) at the apex of the terebra [61]. To assess the full suitability of the host’s internal regions, the wasp must be able to reach into the various regions of its body. Various types of parasitoid wasps have therefore developed a variety of steering mechanisms, including terebra bending and rotation (cf. [32, 56, 62, 63]). Nevertheless, and with respect to the observed behaviour of *M. flavus*, we think that the adjustment of the angle and the depth of insertion of the terebra is not only provided by the steering of the terebra, but mainly by changing the position of the metasoma (and thus also that of the terebra) relative to the site of insertion (Additional file 1, min. 1:27–1:40). An active steering movement of the terebra has recently been observed in the pteromalid wasp *L. distinguendus* [32]. Cervenik et al. [56] have observed a comparable technique in a braconid wasp that involves a passive bending mechanism of the terebra in various directions, achieved by alternating stabbing movements of the 1st valvulae. Therefore, an actively actuated bending movement of the terebra inside the host’s body cannot be excluded for *M. flavus,* especially since we have been unable to observe the actions of the terebra conducted within the host with high-resolution videography. We have been unable to determine the exact moment of venom delivery into the host. However, this process is usually conducted quickly, so that the wasp might simply execute the envenomation on target organs during the process of host assessment. Envenomation can be used either to kill the eggs or larvae of rival wasps previously deposited into the host [64, 65] or to affect the host directly by causing permanent paralysis [18]. Since *M. flavus* is an idiobiont endoparasitoid, it uses its venom to immobilise the host permanently, but without killing it [34]. In none of the observations could we observe any serious form of physical resistance by the host against the wasps, suggesting that the main purpose of paralysis is to halt any further development of the host.

**Egg deposition:** In the following stage, the wasp remains in a stable position, while performing rhythmic contractions of parts of its metasoma (Additional file 1, min. 1:40–1:51). These motions probably represent the initiation of the egg laying process. The highly deformable egg is thus pressed into and passes along the narrow egg canal by alternate back-and-forth movements of the 1st valvulae and with the help of the distally directed ctenidia (ct; Fig. 3d). This process takes place at high speed, just before the wasp extracts its terebra from the host. The rapid valvulae movements cause the entire body of the wasp to be set into vibration (Additional file 1, min. 1:52–2:06). The position in which this is conducted indicates that the egg is deposited immediately beneath the dorsal tergum of the host. Whilst withdrawing the terebra, the wasp secretes a liquid from the terebra’s apex; this liquid hardens when it comes into contact with the air (cf. [40]) putatively forming a so-called feeding tube (Fig. 2f; Additional file 1, min. 2:00–2:10). Indeed, the wasp probably constantly secretes fluids from the terebra apex during the entire drilling and egg laying process (cf. [32]). Such fluids may act as lubricants that provide a cooling effect on the terebra elements during the drilling phase. Additionally, they might prevent the ingress of particles into the terebra [66, 67]. The observed wasps did not use the tube for feeding on the host but left the host after finishing oviposition, suggesting that this structure is formed not only in intended events, but also as a normal byproduct through the hardening of the secreted fluids.

**Host feeding:** In addition to the host’s function as an egg-laying site, the female wasp also needs its host to provide nutrients necessary for egg production [53, 68–70]. Bartlett [68] conducted an experimental study on the host feeding behaviour of *Microterys flavus,* whereas Rosenheim and David [53] investigated this behaviour in another chalcidoid wasp, i.e. *Aphytis lingnanensis* Compere, 1955 (Chalcidoidea: Aphelinidae). In both experiments, the female wasp had to decide whether to use an encountered host for oviposition or as a source of nutrition. This decision is often based on the suitability of the host organism for oviposition, whereby host’s that are considered too small are preferentially used as food source [53, 68]. Bartlett [68] further states that female *M. flavus* only perform host-feeding after their ovipositional capacity and egg supply are depleted, and that host-fed individuals can restore the number of available ripe eggs after a few days. In addition to egg maturation, host feeding also seems to play a role in increasing the overall longevity of the wasp [69, 71]. If the wasp has decided to use the host as a source of nutrition, it also needs to access the host by first piercing its cuticle. The wasp mainly receives nutrition by feeding on the host’s haemolymph (Additional file 1, min. 2:57–3:15; cf. [53, 69]). Therefore, many species of parasitoid wasps create a feeding tube through which the haemolymph can easily be extracted via the wasp’s mouthparts (Fig. 2h).

### Morphological structure of the ovipositor

**Terebra:** To exploit its full functionality, the individual parts of the terebra (i.e. the 1st and 2nd valvulae) must be individually movable, but still form a stable complex. This is achieved by the longitudinal tongue-and-grove-interlocking mechanism called the olistheter. The rhachis is carried ventrally on each side of the unpaired 2nd valvula, whereas the complementary aulax (groove) runs dorsally along both 1st valvulae (rh; au; cf. Fig. 3b). This mechanism allows a longitudinal back-and-forth movement of the elements relative to each other, while preventing their separation. As the terebra of parasitoid wasps lacks any intrinsic musculature, various species have evolved different techniques of terebra steering, involving bending and rotation. The underlying working principles of terebra steering have been intensely studied in selected taxa of Pteromalidae [32], Figitidae [47], Braconidae [5, 56, 62, 63], and Ichneumonidae [5, 63], and Aulacidae and Gasteruptiidae [62] (a general review is given by [57]).

Because of the limited resolution of the SR-µCT images and a lack of histological sections through the terebra of *M. flavus*, a clear assessment of the construction and (transversal) shape of the 2nd valvula and its elements is difficult. However, based on the available data, we assume that the 2nd valvula has become secondarily separated into two halves that overlap asymmetrically at the proximal section of their lengths (2vv; cf. Fig. 9c) and are only fused distally at the very apex. Such specialization of 2nd valvula morphology has also been found in other Chalcidoidea [19, 21, 22, 32], indicating that this trait represents an autapomorphy for the superfamily. In the proximal section of the terebra, both 2nd valvula halves are probably connected by a membrane called the notal membrane [17–22, 24, 25, 32], which transforms into the laminated bridge at the area of the bulbs (blb; Figs. 8a–b, 10a, d) [19, 21, 22, 32]. This specialized morphology is considered to enable the considerable distortions and extensions of the egg canal during oviposition [4, 32, 72].

The medially situated interlock of the 1st valvulae (il1; Fig. 4c) is part of an olistheter-like interlocking mechanism that connects both the 1st valvulae around the area of their apices (cf. [73]). It mainly functions in increasing the stability of the apex during the piercing and drilling of the host's cuticle [5, 32]. A similar structure has been found by Eggs et al. [32] in the pteromalid *L. distinguendus* and by Quicke et al. [5, 8], van Meer et al. [48], Csader et al. [49], and Dweck et al. [73] in various braconids. Similar to the conclusions drawn from observations of *L. distinguendus* [32], the connection of the apices of both the 1st valvulae presumably prevents the distortion of the terebra in its apical region to ensure that the egg is pressed out ventrally between the 1st valvulae, proximally to the interlocking region. The distal notch of the 1st valvula (dn1; Figs. 3a, 4c), located further distally, probably directs various fluids (e.g. venom, lubricants) passed through the terebra to the targeted region.

The dorsomedial surface of both the 1st valvulae that are part of the egg canal is covered with distally directed ctenidia (ct; Fig. 3d) throughout the terebra length. The functionality of the ctenidia probably lies in hooking onto the egg’s chorion and dragging it through the terebra under extreme deformation, actuated by alternating movements of the 1st valvulae, whilst also preventing the egg from moving backwards [74, 75].

**3rd valvulae:** In addition to the previously addressed potential sensory function of the 3rd valvulae in guiding the terebra to the desired drilling site and in preventing a buckling of the terebra during the initial puncturing [5, 32], they probably act as an ovipositor sheath that covers the terebra and provides protection in its resting position [5, 7]. They may further function as an additional mechanical stabilizer during oviposition [48, 56].

**1st valvifer:** Various chalcidoid wasps feature an elongated and bow-shaped 1st valvifer [18–22, 25]. The 1st valvifers of *M. flavus*, however, are only slightly elongated and have a triangular form. Since the rotation of the 1st valvifer is transferred onto the 1st valvulae through the dorsal ramus, factors such as the size, form, and range of motion of the 1st valvifer subsequently determine the distance that the 1st valvulae can be pro- and retracted. Copland [17] conducted a comparative study on the dorsal post-ramus extension across various genera of the Aphelinidae family, since this extension influences the arc of movement that the 1st valvifer can execute. By comparing the overall form of the 1st valvifer between different hymenopteran families, the 1st valvifers of the ichneumonid wasp *Venturia canescens* (Gravenhorst, 1829) [50] or the braconid *Habrobracon hebetor* (Say, 1836) [49] seem to have an oblong shape with rounded edges. Representatives of the Ceraphronoidea show an even stronger elongated and almost rectangularly shaped 1st valvifer [45]. This diversity within 1st valvifer shapes suggests a decisive influence on host selection and the nature of the interaction of wasp and host. A functional lever model based on the species-specific morphology of the 1st valvifer in *M. flavus* is discussed in detail in the paragraph ‘Protraction and retraction of the 1st valvulae’ below. Ctenidia-like structures can be found along the medial surface of the dorsal ramus (ct1; Fig. 6b). By reducing the contact surface between the ramus and the 2nd valvifer, they probably serve to decrease the friction forces between the structures while also minimising the risk of the structures adhering to each other [75].

**2nd valvifer:** Two patches of multiple sensilla are located around the anterior part of the 2nd valvifer. During penetration and egg deposition, they probably have a monitoring function by controlling the movement of the 1st valvifer and its respective position to avoid damage through overextension [16, 17]. Two sensilla (sp; Fig. 5a, d) emerge just ventrally to the intervalvifer articulation, whereas a row of 10 sensilla (sr; Figs. 5a, 6c) is arrayed along the dorsal projection of the 2nd valvifer. Despite minor variations in the number of sensilla, this distribution is common among most of the chalcidoid members, for instance, as observed in Agaonidae [25], Aphelinidae [17], Chalcididae [21], Eurytomidae [20], Pteromalidae [22, 32], and Torymidae [18].

**Female T9:** The female T9 of *M. flavus* carries multiple cuticular projections and apodemes, which mainly function as muscle attachment sites. The posterior end of its exterior surface is scattered with setae, of which some might serve as sensory organs for mechanoreception (cf. [16]).

### Mechanics and mode of function of the ovipositor system

Because of the morphological features of the individual cuticular elements of the ovipositor system, allowing them to move relative to each other and to transmit motion through multiple articulations, the musculoskeletal ovipositor system of *M. flavus* can perform several targeted actions that are related to accessing the host for oviposition. These mainly include the motion (depression, elevation, and rotation) of the whole terebra and the pro- and retractional movements of the 1st valvulae. The ovipositor muscles actuate the various movements enabling a successful oviposition. In *M. flavus*, two pairs of two muscles (m-a-2vf-2vv & m-p-2vf-2vv; m-d-T9-2vf-a/b & m-v-T9-2vf) work antagonistically during ovipositor motion, whereas three muscles (m-1vf-gm; m-p-T9-2vf; m-T9-gm) have a stabilizing effect on the ovipositor system.

**Depression and elevation of the terebra/bending of the terebra:** The majority of parasitoid hymenopterans studied so far use the paired posterior 2nd valvifer-2nd valvula muscles to depress the terebra into an active probing position and their antagonistically working anterior 2nd valvifer-2nd valvula muscles to elevate the terebra back towards the resting position after an oviposition attempt [6, 16, 45, 48, 50, 76, 77]. Both muscles insert around the bulbs of the 2nd valvula. The **posterior 2nd valvifer-2nd valvula muscle** (m-p-2vf-2vv; Fig. 7d–f, 8a–b) exerts a pulling force on the processus musculares (prm; Fig. 8c), which emerges dorsally from the anteriorly directed horn-like structures of the bulbs of the 2nd valvula. This causes the 2nd valvulae and the interlocked 1st valvulae to be depressed, i.e. rotated downwards towards an active probing position, until the terebra is oriented almost perpendicularly to the abdomen [6, 45, 48, 50]. Antagonistically, the **anterior 2nd valvifer-2nd valvula muscle** (m-a-2vf-2vv; Figs. 7d–f, 8a–b) pulls the processus articularis (pra; Fig. 8c–d), located laterally at the bulbs of the 2nd valvulae, upwards. This leads to a rotation of the 2nd and 1st valvulae into a dorsal direction around the basal articulation [48, 50] and the elevation of the terebra into its resting position back between the 3rd valvulae. Copland and King have postulated a similar mechanism for chalcidoid species of Aphelinidae [17] and Mymaridae [23].

However, in both the chalcidoid species recently investigated, namely the pteromalid *L. distinguendus* [32] and the encyrtid *M. flavus* in the present study, the depression of the terebra has been observed to be exclusively achieved through a downward bending of the full metasoma, instead of through an isolated depression of the terebra itself. This behaviour has also been reported for other species of Pteromalidae [40, 78], Torymidae [18], Eurytomidae [20], and Eulophidae [19]. Therefore, we assume that the m-p-2vf-2vv in *M. flavus* and other chalcidoids only acts as supporting muscle or even has no influence at all on lowering the terebra into a drilling position. It presumably is adapted in its main function (see paragraph ‘Rotation of the terebra’ below; cf. [32]). The m-p-2vf-2vv can still be actively used to cause the elevation of the terebra back into resting position. In addition to preparing the terebra for drilling, the muscles’ ability to elevate and depress the terebra may also be used to support its bending movements within the host, thus aiding internal host assessment (cf. [32]).

**Rotation of the terebra:** In addition to elevation and depression, the **posterior** and the **anterior 2nd valvifer-2nd valvula muscle** are probably also used to perform rotational movement of the terebra around its longitudinal axis. These muscles have previously been associated with terebra rotation for representatives of Pteromalidae [40, 78], Torymidae [18], Eurytomidae [20], Tetracampidae, and Eulophidae [19]. Eggs et al. [32] were able to clearly demonstrate the rotational terebra movement in the pteromalid wasp *L. distinguendus*, whereas Vincent [58] observed it during ovipositor drilling in wood wasps. A contraction of the posterior 2nd valvifer-2nd valvula muscle on one side will presumably cause the terebra to rotate to a certain degree. Contraction of the right m-p-2vf-2vv causes a clockwise rotation of the terebra, whereas a contraction of the left m-p-2vf-2vv causes its anticlockwise rotation. These rotational movements might be further supported by the contractions of the m-a-2vf-2vv muscles [32]. When the terebra is anchored into the host’s dorsal shield, *M. flavus* wasps presumably combine rotational terebra movements with stabbing movements of the individual valvulae to initiate the penetration of the host’s dorsum (cf. [32]).

**Protraction and retraction of the 1st valvulae:** The terebra consists of three individual parts (paired 1st valvulae; Figs. 3, 4a, c; unpaired 2nd valvulae; Figs. 3, 9c) that are connected by the olistheter. Thus, each of the paired 1st valvulae can be independently pro- and retracted in relation to the 2nd valvula. This movement is actuated by **part a and b** of the **dorsal T9-2nd valvifer muscle** (m-d-T9-2vf-a/m-d-T9-2vf-b; Fig. 7d–f; summarized as *F*_m-d-T9-2vf_; Fig. 8e) and the antagonistically working **ventral T9-2nd valvifer muscle** (m-v-T9-2vf; Fig. 7d–f; summarized as *F*_m-v-T9-2vf_; Fig. 8e). The complex of the dorsal T9-2nd valvifer muscle connects the posterior end of the female T9 with the anteriorly situated dorsal lobe of the 2nd valvifer. Upon contraction of the m-d-T9-2vf (*F*_m-d-T9-2vf_; Fig. 8e), the female T9 and the 2nd valvifer are pulled towards each other, whereby both elements exert an opposite force on the intermediately situated 1st valvifer via the two rotational joints (intervalvifer and tergo-valvifer articulation) that connect the female T9 and the 2nd valvifer to the 1st valvifer. This results in an anteriorly directed rotation of the 1st valvifer around the intervalvifer articulation [6, 32, 45, 48, 50]. The 1st valvifer hereby acts as a one-armed lever that transmits the rotational force via the dorsal ramus onto the 1st valvula, causing it to slide distally relative to the 2nd valvula [32, 48, 50]. The ventral T9-2nd valvifer muscle works antagonistically against this motion, causing an opposite sequence of movements. It connects the anterior cordate apodeme of the female T9 with the posterior part of the dorsal flange of the 2nd valvifer, causing both elements to move apart from each other when contracted (*F*_m-v-T9-2vf_; Fig. 8e). Opposite to the previously described movement, the 1st valvifer is now rotated posteriorly, which results in a posteriorly directed retraction of the 1st valvulae relative to the 2nd valvula. To convert this motion into the penetration of the substrate and/or the host’s cuticle, the muscles on each side of the ovipositor apparatus work alternately, causing an alternate back-and-forth sliding of each 1st valvula [16, 45, 48, 50, 56, 57] to initiate the previously described ‘push–pull’ mechanism [57–59]. To simplify the estimation of the torques (*M*) generated by the contractions of the dorsal and ventral T9-2nd valvifer muscles (*F*_m-d-T9-2vf_/*F*_m-v-T9-2vf_; Fig. 8e), the following assumptions have been made: (1) the 2nd valvifer serves as the reference frame, making the intervalvifer articulation (iva; Figs. 5a, d, 8e) the pivot point around which the 1st valvifer rotates; (2) the musculoskeletal system constraints the movements of the 2nd valvifer and the female T9, thereby preventing any rotation of the two elements around the articulations and only allowing telescopic sliding along the anterior–posterior axis; and (3) frictional forces in the system can be neglected. Under these assumptions, the horizontal force vector components act on the 1st valvifer at the tergo-valvifer articulation (tva; Figs. 5a, c, 8e) in the anterior–posterior axis (*F*_m-d-T9-2vf(x)-in_/*F*_m-v-T9-2vf(x)-in_; Fig. 8e). Consequently, the torques (*M*) of *F*_m-d-T9-2vf_ and* F*_m-v-T9-2vf_ can be estimated using the following variables: (1) the horizontal vector components (*F*_m-d-T9-2vf(x)-in_/*F*_m-v-T9-2vf(x)-in_; Fig. 8e) of the maximum force of a muscle, (2) the anatomical inlever arm (a; Fig. 8e), defined by the distance between the intervalvifer and the tergo-valvifer articulation, and (3) the joint angle (*α*; Fig. 8e). The calculation can be carried out according to the following equations:

1 $$M_{{\text{m-d-T9-2vf}}} = F_{{{\text{m-d-T9-2vf}}\left( {\text{x}} \right){\text{-in}}}} \cdot \mathrm{a} \cdot \sin \left( \alpha \right)$$

2 $$M_{{\text{m-v-T9-2vf}}} = F_{{{\text{m-v-T9-2vf}}\left( {\text{x}} \right){\text{-in}}}} \cdot \mathrm{a} \cdot \sin \left( \alpha \right)$$

In combination with the anatomical inlever (a; Fig. 8e) and the anatomical outlever arm (b; Fig. 8e), the 1st valvifer acts as a one-arm class 3 lever, whereby the anatomical outlever is defined by the distance between the intervalvifer articulation and the point at which the 1st valvifer merges into the dorsal ramus of the 1st valvulae. The resulting pro- and retracting forces acting at the dorsal ramus of the 1st valvulae (*F*_m-d-T9-2vf-out_ */F*_m-v-T9-2vf-out_; Fig. 8e) can be estimated using the following variables: (1) the horizontal vector components (*F*_m-d-T9-2vf(x)-in_/*F*_m-v-T9-2vf(x)-in_; Fig. 8e) of the forces acting on the 1st valvifer at the tergo-valvifer articulation, (2) the length of the effective inlever (a′ = a · sin(α); Fig. 8e), and (3) the effective outlever arm (b′ = b · sin(*β*); Fig. 8e). The calculation can be carried out according to the following equations:

3 $$F_{{\text{m-d-T9-2vf-out}}} = \left( {F_{{{\text{m-d-T9-2vf}}\left( {\text{x}} \right){\text{-in}}}} \cdot \, \mathrm{a^\prime}} \right)/ \, \mathrm{b^\prime}$$

4 $$F_{{\text{m-v-T9-2vf-out}}} = \left( {F_{{{\text{m-v-T9-2vf}}\left( {\text{x}} \right){\text{-in}}}} \cdot \, \mathrm{a^\prime}} \right)/ \, \mathrm{b^\prime}$$

The overall shape of the 1st valvifer and the respective positions of the intervalvifer and tergo-valvifer articulation vary among the hymenopteran families (cf. [32, 45, 49, 50]). The ratio of the effective outlever to the effective inlever (b′/a′ ratio) is lower than that observed in the pteromalid *L. distinguendus* [32]. This morphological feature suggests that, in *M. flavus*, the speed and mechanical deflection of the 1st valvula is slightly reduced in favour of maximum force output (cf. [32, 48, 49]). An even stronger focus on maximising the force output has presumably been observed in the braconid *Habrobracon hebetor* [49], where the effective outlever almost equals the effective inlever.

Medially emerging sawteeth at the apex of the 2nd valvula help to cut or anchor the terebra elements into the substrate or the host’s skin (cf. [32, 72]). An additional function of the pro- and retraction of the 1st valvulae involves helping an egg pass through the much narrower egg canal for egg deposition. Together with the distally directed ctenidia (ct; Fig. 3d), the alternating back-and-forth motions of the 1st valvulae drag the egg through the terebra under extreme deformation [74, 75]. From their work on the pteromalid wasp *Nasonia vitripennis* (Walker, 1836), King and Rafai [79] showed that the deformation of the egg’s shape does not harm it but is a necessary stimulus to induce embryonic development.

**Stabilization of the ovipositor:** Depending on the environment, the ovipositor system must meet certain requirements in terms of stability and mechanical stress resistance. In general, the need to reach a concealed host requires the ovipositor to perform intense drilling motions and to overcome the physical resistance exerted by the substrate [45]. Furthermore, the penetration of an exposed host requires the oviposition procedure and thus the motion of the individual elements to be conducted rapidly, as the host is likely to perform escape movements. Throughout oviposition, three individual muscles presumably mainly act as tensor muscles in a stabilizing way, two of them inserting at the genital membrane.

One of these muscles is the **1st valvifer-genital membrane muscle** (m-1vf-gm; Figs. 7d–f, 9a–c), which originates at the posteriorly situated interarticular ridge of the 1st valvifer, immediately between the intervalvifer and the tergo-valvifer articulation. This area approximately corresponds to the rotation axis of the 1st valvifer. Thus, this muscle probably serves both to stabilize the 1st valvifer and to fixate it in its position during rotation. Two further stabilizing muscles are the **T9-genital membrane muscle** (m-T9-gm; Figs. 7d–f, 9d, e) and the **posterior T9-2nd valvifer muscle** (m-p-T9-2vf; Figs. 7d–f, 9f, g), both originating at the posterodorsal region of the female T9. The two muscles presumably hold the female T9 and the 2nd valvifer in their position and prevent them from rotating around the articulations that connect them to the 1st valvifer [16, 32, 50]. All three stabilizing muscles have only recently been described for Chalcidoidea, namely for the pteromalid *L. distinguendus* [32], but not yet for encyrtids.

Historically, these muscles were probably overlooked because of a combination of their minute size and the invasive methods of dissection used in the previous studies of Copland [17] and Copland and King [18, 20–23, 25]. Moreover, the T9-genital membrane muscle arises in the direct proximity of the posterior T9-2nd valvifer muscle, possibly leading to the false interpretation of the two as a single muscle, instead of two distinct ones.

**Support of the venom and reproductive system:** The **dorsal** and **ventral 2nd valvifer-venom gland reservoir muscles** (m-d-2vf-vr-a/b & m-v-2vf-vr-a/b) are found medially on the anterior-most part of the 2nd valvifer (see Figs. 7d–f, 8a–b,). Their potential function has previously been discussed for pteromalid wasps [32] and various ant species [42–44]. Quicke [5, 7] provides a basic overview on the internal reproductive anatomy of adult parasitoid wasps, whereas Copland [17] and Copland and King [18–23, 25] specify this for various representatives of chalcidoids. According to these authors, the female genital system bears a dorsally situated pair of ovarioles that open into a pair of lateral oviducts, which then fuse into the common oviduct. Between both ovipositor halves and posteroventral to the common oviduct lies the prominent venom apparatus, consisting of a posteriorly located venom gland and the venom gland reservoir (vr; Fig. 9a–c). The venom enters the common oviduct just prior to the terebra bulbs via the orifice of the venom gland reservoir (cf. [18, 32]). As shown for an eulophid wasp, the eggs are led towards the ovipositor base via the common oviduct, where the egg is coated with a lubricant by glandular cells [80] and/or a layer that protects it from the host’s immune reaction [81]. This also includes the Dufour’s gland (= alkaline gland sensu [17–22, 25, 41]), which lies dorsolateral to the venom reservoir and enters into the common oviduct, as it has been shown for the pteromalid *L. distinguendus* [32]. The fluids secreted by the Dufour’s gland show highly diverse functions in hymenopterans but are hypothesized to be used for communication and to mark the host individual in parasitoid wasps [82]. Although not visible in the observed material, we expect a similar gland to be present in *M. flavus*.

We hypothesize that the dorsal and ventral 2nd valvifer-venom gland reservoir muscles connect the 2nd valvifer with both the venom gland reservoir and its orifice. The **dorsal 2nd valvifer-venom gland reservoir muscle** (m-d-2vf-vr; Figs. 7d–f, 8a–b, 10a–c) inserts dorsally at the anterior part of the venom gland reservoir at a membrane ventral to the common oviduct (co; Fig. 10). A contraction of this muscle probably helps to discharge secretions from the venom gland and presumably also from the Dufour’s gland into the common oviduct and subsequently into the egg canal. In addition, this muscle might act as a tensor muscle that stabilizes the 2nd valvifer during movement of the ovipositor (cf. [32]). Because of its insertion at a membranous layer that directly borders the common oviduct, another function of the m-d-2vfvr might lie in expanding the common oviduct, thereby aiding the controlled transfer of an egg into the egg canal of the terebra.

**Part A** and **part B** of the **ventral 2nd valvifer-venom gland reservoir muscle** (m-v-2vf-vr-a/b; Figs. 7d–f, 8a–b, 10d–i) both insert laterally at the orifice of the venom gland reservoir (ovr; Fig. 10d–i) immediately before its entrance into the common oviduct. Upon contraction, they might open up a passage and enable the controlled discharge of venom and probably of Dufour’s gland secretion into the common oviduct and subsequently the egg canal (cf. [32]).

## Conclusions

The present study represents the first thorough description of the oviposition behaviour and the musculoskeletal ovipositor system in an encyrtid wasp, namely *Microterys flavus*, a parasitoid of scale insects. We describe and discuss all the cuticular elements of the ovipositor and the set of nine paired ovipositor muscles that actuate the various ovipositor movements, including the elevation and depression of the terebra and drilling through the substrate or the host cuticle. The ovipositor features the same basic composition of cuticular elements across all examined hymenopteran taxa (three pairs of valvula, two pairs of valvifers, and the female T9). However, morphological adaptations of the terebra (i.e. a longitudinally split 2nd valvula that is fused only at the apex) combined with functional adaptations of the anterior and the posterior 2nd valvifer-2nd valvula muscles presumably enabled *M. flavus*, and potentially chalcidoid wasps in general, to perform some terebra bending and rotational movements. These terebra movements are crucial for penetrating the host’s cuticle, injecting venom, assessing the host’s interior, and accurately depositing eggs. In addition, we describe two paired muscles (namely, the dorsal and the ventral 2nd valvifer-venom gland reservoir muscles) that have been overlooked in most previous studies. These muscles are associated with the venom gland reservoir, potentially supporting the controlled outflow of venom or other secretions for egg laying and host manipulation. This study is part of a series of studies in which synchrotron-µCT data have been used to create detailed 3D models of the musculoskeletal ovipositor system of parasitoid wasps, including functional aspects of the oviposition process [32, 48, 50]. These models, together with microscopical analyses and behavioural observations, provide a foundation for morpho-physiological, behavioural, and ecological comparisons across different groups of parasitoid wasps. They also add to our understanding of a putative key feature that has largely contributed to the high evolutionary success of Chalcidoidea (cf. [2, 32, 83]).

The hymenopteran terebra and that of chalcidoids, in particular, can act as suitable biological concept generators and, thus, their further investigation might be helpful for the development and design of slender man-made probing tools, e.g. for micro-invasive surgery (cf. [84–88]).

## Methods

The *M. flavus* individuals used in this study were acquired from Sautter & Stepper GmbH (Ammerbuch, Germany). Approximately half of the wasps were killed in 70% ethanol, whereas the other half was kept alive and fed with watered honey for a few days up until the in vivo documentation of its oviposition behaviour. During this process, the wasps were kept in a plastic container with an oxygen-permeable stopper at constant room temperature. The lateral habitus image of *M. flavus* was obtained with a digital microscope (Keyence Digital Microscope VHX-7000, Keyence Corporation, Osaka, Japan) by using focus stacking. All used images were pre-processed (white/black balancing, cropping) with GIMP version 2.10.30 (https://www.gimp.org; RRID:SCR_003182) and then compiled and edited with Inkscape version 1.1 (https://www.inkscape. org; RRID:SCR_014479).

### High-resolution videography

A total of five behavioural recordings were conducted to capture the act of oviposition and to define the different stages of the process. We released five different females of *M. flavus* each together with a single individual of *Coccus hesperidum* into a 5 · 4 · 3 mm closed chamber. The chamber itself was composed of Plexiglass to ensure the best possible light transmission and a bright environment for recording. The front and top openings of the chamber were sealed each with a clean glass cover slide. The process was filmed by a Nikon D7100 single lens reflex digital camera (Nikon Corporation, Tokyo, Japan) with the software Helicon Remote version 3.6.2.w (Helicon Soft Ltd, Kharkiv, Ukraine) mounted onto a Leica MZ 12.5 stereomicroscope (Leica Microsystems GmbH, Wetzlar, Germany) fitted with two LED cold-light sources KL 300 LED (Schott AG, Jena, Germany). The focus was adjusted manually.

### Scanning electron microscopy (SEM)

20 individuals of *M. flavus* were killed in 70% ethanol at room temperature, dissected under a binocular by using fine forceps and prepared for drying. Another 15 individuals were killed with the same method but, here, the metasomas were separated from the bodies and macerated in 10% aqueous potassium hydroxide (KOH) for roughly 12 h, washed in distilled water and prepared for subsequent drying. The ovipositors obtained from both approaches were dehydrated in a graded ethanol (C_2_H_6_O) series (30, 50, 70, 80, 90, 95, once each, and twice in 100%, for 30 min at each concentration) and critical-point-dried using a Polaron 3100 (Quorum Technologies Ltd, East Sussex, UK). To analyse the objects in an SEM, they were mounted onto stubs with double-sided adhesive carbon tape and sputter-coated with a 19-nm-thick layer of pure gold (AU) by using an Emitech K550X (Quorum Technologies Ltd, East Sussex, UK). Investigation and imaging were performed with a Zeiss EVO LS 10 SEM (Carl Zeiss Microscopy GmbH, Jena, Germany) controlled with the software SmartSEM version V05.04.05.00 (Carl Zeiss Microscopy GmbH, Jena, Germany).

### Synchrotron X-ray phase-contrast microtomography (SR-µCT)

Being a non-invasive technique, SR-µCT has the advantage of avoiding the destruction of an organism by preparation and leaving the sought-after organ in its natural position [89]. Furthermore, it enables 3D modelling at sub-micrometre resolution and, thus, the illustration of complex anatomical features [89]. The ethanol-fixed metasoma of a female *M. flavus* was dehydrated stepwise in ethanol and dried through critical point drying by using a Polaron 3100 (Quorum Technologies Ltd., East Sussex, UK). The anterior end of the metasoma was then glued onto plastic pins and subsequently mounted onto the goniometer head of the sample stage. Microtomography scans were acquired using the 150 m-long imaging beamline ID19 [89] of the European Synchrotron Radiation Facility (ESRF) in Grenoble (France) by using a photon energy of 19.5 keV (wavelength 8 · 10^–11^ m) and an effective detector pixel size of 0.32 μm resulting in a corresponding field of view of 0.63 · 0.63 mm^2^. The indirect high-resolution X-ray imaging detector consisted of a 4.5 µm thick LSO:Tb (Tb-doped Lu_2_SiO_5_) single-crystal scintillator lens (magnification 20×, numerical aperture (NA) 0.4) coupled to a sCMOS-based camera type pco.edge 5.5 (2560 × 2160 pixels, 6.5 μm pixel size, Excelitas PCO GmbH, Kehlheim, Germany) [90, 91]. The detector-to-sample distance was set to 10 mm to benefit from propagation-based X-ray phase contrast for enhanced imaging sensitivity. A total of 6000 projections were recorded over the sample rotation of 180° around the axis of the plastic pin [89]. To boost the contrast further, single-distance phase retrieval was applied to the recorded 2D radiographs by using a so-called modified Paganin-approach with manually trimmed values for the required complex-valued index of refraction parameters [92, 93]. Based on the processed 2D radiographs, a 3D dataset was reconstructed using the filtered back-projection algorithm [94, 95] developed for parallel-beam tomography based on ESRF-in-house developed software.

The two resulting tomograms were registered and calibrated in Fiji [96] (https://imagej.net/Fiji; RRID:SCR_002285), an image processing package for the software ImageJ version 1.51d ([97, 98]; https://imagej.net), and subsequently imported to the plugin TrakEM2 [99] (RRID:SCR_008954) for stitching and cropping. Pre-segmentation was accomplished with Amira version 6.0 (FEI Company, Hillsboro, OR, USA; RRID:SCR_014305). The various cuticular structures and the ovipositor muscles were labelled manually in approximately every 25th virtual slice. These labels then served as input for the automated segmentation by using the Biomedical Image Segmentation App ‘Biomedisa’ [100] (https://biomedisa.org). After some minor manual corrections of the segmentation results of the ‘Biomedisa’ output by using Amira, we converted them into polygon meshes with some minor smoothing (unconstrained smoothing; smoothing extent: 2) and polygon reduction to create a full 3D model (surface mesh).

## Supplementary Information

**Additional file 1 **Video sequences of female *Microterys flavus*, parasitising its host organism *Coccus hesperidum* in an artificial film chamber (cf. Fig. 2). The video shows the drilling process, the egg deposition and a host feeding event. Part 1: The oviposition process; Part 2: Host feeding. (MP4 17694 KB)

**Additional file 2** Animation of the rotating segmented 3D model of the musculoskeletal ovipositor system of *Microterys flavus* (cf. Figs. 7–10). (MP4 192536 KB)

## Data Availability

All data supporting the conclusions of this article are included within the article and its additional files. The full resolution videos (Additional file 1, Additional file 2) and the analysed raw datasets are available from the corresponding author on reasonable request.

## Appendix 1

See Table 3.

**Table 3:** Morphological terms relevant to the hymenopteran ovipositor system. The terms are used and defined according to the Hymenoptera Anatomy Ontology (HAO) [37–39]; the respective Uniform Resource Identifiers (URI) and the synonyms found in the cited literature are listed

Anatomical term (abbreviation)	Definition/concept	Synonyms commonly found in literature	URI
1st valvifer (1vf)	The area of the 1st valvifer-1st valvula complex that is proximal to the aulax, bears the 9th tergal condyle of the 1st valvifer and the 2nd valviferal condyle of the 1st valvifer and is connected to the genital membrane by muscle	1. Valvifer; Fulcral plate; Furcal plate; Gonangulum, Gonangula; Gonocoxite 8; Gonocoxite XIII; Triangular plate; Vorderer Valvifer; Winkelplatte	http://purl.obolibrary.org/obo/HAO_0000338
1st valvifer-1st valvula complex	The anatomical cluster composed of the sclerites, articulating with the 9th abdominal tergite and the 2nd valvifer		http://purl.obolibrary.org/obo/HAO_0002158
1st valvifer-2nd valvifer muscle	The ovipositor muscle that arises from the interarticular ridge of the 1st valvifer and inserts on the 2nd valvifer		http://purl.obolibrary.org/obo/HAO_0002189
1st valvifer-genital membrane muscle (m-1vf-gm)	The ovipositor muscle that arises from the posterior part of the 1st valvifer and inserts anteriorly on the genital membrane anterior to the T9-genital membrane muscle		http://purl.obolibrary.org/obo/HAO_0001746
1st valvula (1vv)	The area of the 1st valvifer-1st valvula complex that is delimited distally by the proximal margin of the aulax	Gonapophysis 8; Gonapophysis VIII; Lancet; Lower valve; Stechborste; Stylet; Ventral valve	http://purl.obolibrary.org/obo/HAO_0000339
2nd valvifer (2vf)	The area of the 2nd valvifer-2nd valvula-3rd valvula complex that is proximal to the basal articulation and to the processus musculares and articulates with the female T9	Gonocoxite 9; Gonocoxite IX; Inner plate; ~ Inner ovipositor plate; Oblong plate; Oblonge Platte; ~ Semicuricular sheet	http://purl.obolibrary.org/obo/HAO_0000927
2nd valvifer-2nd valvula-3rd valvula complex	The area that is connected to the 9th tergite and the 1st valvifer via the conjunctiva, is articulated to the 1st tergite and bears the aulax		http://purl.obolibrary.org/obo/HAO_0002175
2nd valvifer-3rd valvula complex	The area of the 2nd valvifer-2nd valvula-3rd valvula complex that is proximal to the basal articulation		http://purl.obolibrary.org/obo/HAO_0002181
2nd valvula (2vv)	The area of the 2nd valvifer-2nd valvula-3rd valvula complex that is distal to the basal articulation and to the processus musculares and is limited medially by the median body axis	Dorsal valve; Gonapophysis 9; Gonapophysis IX; Sheath; Stylet sheath; Upper valve	http://purl.obolibrary.org/obo/HAO_0000928
3rd valvula (3vv)	The area of the 2nd valvifer-3rd valvula complex that is posterior to the distal vertical conjunctiva of the 2nd valvifer3rd valvula complex	Articulating palps; Gonostylus; Gonostylus IX; ~ Inner ovipositor plate; Ovipositor sheath ; Palp; Sheath lobe; Terminal palp	http://purl.obolibrary.org/obo/HAO_0001012
Anterior 2nd valvifer-2nd valvula muscle (m-a-2vf-2vv)	The ovipositor muscle that arises from the anterodorsal part of the 2nd valvifer and inserts subapically on the processus articularis	Anterior gonocoxapophyseal muscle; Gonapophysis 9 levator; Shaft elevator muscle	http://purl.obolibrary.org/obo/HAO_0001166
Anterior angle of the 1st valvifer	The corner of the 1st valvifer that marks the posterior end of the 1st valvula		http://purl.obolibrary.org/obo/HAO_0002168
Anterior flange of abdominal tergum 9 (af9)	The flange that extends along the anterolateral margin of female T9		http://purl.obolibrary.org/obo/HAO_0001171
Anterior ridge of T9	The ridge that extends along the anterior margin of female T9 and receives the site of origin of the ventral and the dorsal T9-2nd valvifer muscles		http://purl.obolibrary.org/obo/HAO_0002182
Anterior section of dorsal flange of the 2nd valvifer	The area of the dorsal flange of the 2nd valvifer that is anterior to the site of origin of the basal line	~ Semicircular sheet	http://purl.obolibrary.org/obo/HAO_0002173
Articular surface	The area that is located on the sclerite and that makes movable direct contact with another sclerite		http://purl.obolibrary.org/obo/HAO_0001485
Aulax (au)	The impression that is on the 1st valvifer-1st valvula complex and accommodates the rhachis	Groove	http://purl.obolibrary.org/obo/HAO_0000152
Basal articulation (ba)	The articulation that is part of the 2nd valvifer-2nd valvula-3rd valvula complex and adjacent to the rhachis	Bulbous articulation	http://purl.obolibrary.org/obo/HAO_0001177
Bulb (blb)	The anterior area of the dorsal valve [composite structure of the fused 2nd valvulae] that is bulbous	Bulbous basal part of the united 2nd valvulae; Boulbous sockets; Pivoting process; Sockets	http://purl.obolibrary.org/obo/HAO_0002177
Common oviduct (co)	The duct that is unpaired and connects the lateral oviducts with the vagina through the gonopore	Median oviduct; Vagina	http://purl.obolibrary.org/obo/HAO_0002601
Cordate apodeme	The apodeme on the anterior margin of female T9. The ventral T9-2nd valvifer muscle attaches partly on the apodeme		http://purl.obolibrary.org/obo/HAO_0001585
Dorsal flange of the 2nd valvifer (df2)	The flange that extends on the dorsal margin of the 2nd valvifer. Part of the ventral T9-2nd valvifer muscle attaches to the flange		http://purl.obolibrary.org/obo/HAO_0001577
Dorsal projection of the 2nd valvifer (dp2)	The projection that is located on the 2nd valvifer and corresponds to the proximal end of the rhachis	~ Ramus edge	http://purl.obolibrary.org/obo/HAO_0002172
Dorsal ramus of the 1st valvula (dr1)	The region that extends along the dorsal margin of the 1st valvula and bears the aulax	1st ramus; Ramus of the 1st valvula	http://purl.obolibrary.org/obo/HAO_0001579
Dorsal T9-2nd valvifer muscle (m-d-T9-2vf-a/b)	The ovipositor muscle that arises along the posterodorsal part of the anterior margin of female T9 and inserts on the anterior section of the dorsal flanges of the 2nd valvifer	Anterior tergogonocoxal muscle; Dorsal/Ventral [= part a/part b] anterior tergal muscle of the 2nd valvifer; Extensor muscles of lancet; Upper/lower [= part a/part b] stylet protractor muscle	http://purl.obolibrary.org/obo/HAO_0001569
Egg canal (ec)	The anatomical space that is between the left and right olistheters		http://purl.obolibrary.org/obo/HAO_0002191
Female T9 (T9)	The tergite that is articulated with the 1st valvifer and is connected to the 2nd valvifer via muscles	9th tergum; Outer ovipositor plate; Outer plate; Quadrate plate; Sled plate; T9; Tergite IX; Tergum 9; Tergum IX	http://purl.obolibrary.org/obo/HAO_0000075
Genital membrane (gm)	The conjunctiva that connects the ventral margins of the 2nd valvifers arching above the 2nd valvula		http://purl.obolibrary.org/obo/HAO_0001757
Integument	The anatomical system that forms the covering layer of the animal, ectodermal in origin and composed of epidermal cells producing the cuticle		http://purl.obolibrary.org/obo/HAO_0000421
Interarticular ridge of the 1st valvifer (iar)	The ridge that extends along the posterior margin of the 1st valvifer between the intervalvifer and tergovalvifer articulations		http://purl.obolibrary.org/obo/HAO_0001562
Intervalvifer articulation (iva)	The articulation between the 1st valvifer and 2nd valvifer		http://purl.obolibrary.org/obo/HAO_0001558
Median bridge of the 2nd valvifers (mb2)	The area that connects posterodorsally the 2nd valvifers and is the site of attachment for the posterior T9-2nd valvifer muscle		http://purl.obolibrary.org/obo/HAO_0001780
Olistheter	The anatomical cluster that is composed of the rhachis of the 2nd valvula and the aulax of the 1st valvula		http://purl.obolibrary.org/obo/HAO_0001103
Orifice of the venom gland reservoir (ovr)	The anatomical space at the proximal-most end of the venom gland reservoir	Opening of the reservoir	http://purl.obolibrary.org/obo/HAO_0002599
Ovipositor	The anatomical cluster that is composed of the 1st valvulae, 2nd valvulae, 3rd valvulae, 1st valvifers, 2nd valvifers and female T9	Ovipositor mechanism	http://purl.obolibrary.org/obo/HAO_0000679
Ovipositor muscle	The abdominal muscle that inserts on the ovipositor		http://purl.obolibrary.org/obo/HAO_0001290
Posterior 2nd valvifer-2nd valvula muscle (m-p-2vf-2vv)	The ovipositor muscle that arises posteroventrally from the 2nd valvifer and inserts on the processus musculares of the 2nd valvula	Gonapophysis 9 depressor; Posterior gonocoxapophyseal muscle	http://purl.obolibrary.org/obo/HAO_0001815
Posterior area of the 2nd valvifer	The area of the 2nd valvifer that is posterior to the anatomical line that is the shortest distance from the valviferal fossa of the 2nd valvifer to the ventral margin of the 2nd valvifer		http://purl.obolibrary.org/obo/HAO_0002170
Posterior section of dorsal flange of the 2nd valvifer	The area of the dorsal flange of the 2nd valvifer that is posterior to the site of origin of the basal line	~ Inner ovipositor plate	http://purl.obolibrary.org/obo/HAO_0002174
Posterior T9-2nd valvifer muscle (m-p-T9-2vf)	The ovipositor muscle that arises medially from the posterodorsal part of female T9 and inserts on the median bridge of the 2nd valvifers	Dorsal/Lateral tergogonostylar muscle	http://purl.obolibrary.org/obo/HAO_0001813
Processus articularis (pra)	The process that extends laterally from the proximal part of the 2nd valvula and forms the median part of the basal articulation, and corresponds to the site of attachment for the anterior 2nd valvifer-2nd valvula muscle		http://purl.obolibrary.org/obo/HAO_0001704
Processus musculares (prm)	The apodeme that extends dorsally from the proximal part of the 2nd valvula to the genital membrane and receives the site of attachment of the posterior 2nd valvifer-2nd valvula muscle		http://purl.obolibrary.org/obo/HAO_0001703
Rhachis (rh)	The ridge that extends along the ventral surface of the 2nd valvula that is partially enclosed by the aulax		http://purl.obolibrary.org/obo/HAO_0000898
Ridge	The apodeme that is elongate		http://purl.obolibrary.org/obo/HAO_0000899
Sawteeth (st)	The process that is located along the ventral margin of the 1st valvula or the dorsal margin of the 2nd valvula	Barb, barbs; Teeth; Sheath teeth	http://purl.obolibrary.org/obo/HAO_0001681
Sclerite	The area of the cuticle that is strongly sclerotized with thick exocuticle and is surrounded by conjunctivae		http://purl.obolibrary.org/obo/HAO_0000909
Sensillar patch of the 2nd valvifer (sp)	The patch that is composed of placoid sensilla adjacent to the intervalvifer articulation		http://purl.obolibrary.org/obo/HAO_0001671
T9-genital membrane muscle (m-T9-gm)	The ovipositor muscle that arises from the cordate apodeme and inserts dorsally on the proximal part of the genital membrane and on the opposite cordate apodeme		http://purl.obolibrary.org/obo/HAO_0001639
Tendon	The portion of tissue that is fibrous and strong, composed of tendon cells and connects the muscle to the integument		http://purl.obolibrary.org/obo/HAO_0000996
Terebra (trb)	The anatomical cluster that is composed of the 1st and 2nd valulae	Ovipositor; (ovipositor) shaft; Shaft of ovipositor	http://purl.obolibrary.org/obo/HAO_0001004
Tergite	The sclerite that is located on the tergum		http://purl.obolibrary.org/obo/HAO_0001005
Tergo-valvifer articulation (tva)	The articulation that is located between the female T9 and the 1st valvifer and is composed of the 9th tergal condyle of the 1st valvifer and the 1st valviferal fossa of the 9th tergite		http://purl.obolibrary.org/obo/HAO_0001636
Venom gland reservoir (vr)	The gland reservoir that is between the 2nd valvifers		http://purl.obolibrary.org/obo/HAO_0002176
Ventral ramus of the 1st valvula	The area that extends external to the dorsal ramus of the 1st valvula		http://purl.obolibrary.org/obo/HAO_0000891
Ventral ramus of the 2nd valvula	The area of the 2nd valvifer-2nd valvula-3rd valvula complex that bears the rhachis		http://purl.obolibrary.org/obo/HAO_0001107
Ventral T9-2nd valvifer muscle (m-v-T9-2vf)	The ovipositor muscle that arises from the lateral region of female T9 and inserts along the posterior part of the dorsal flange of the 2nd valvifer	Gonapophysis 8 retractor; Posterior tergal muscle of the 2nd valvifer; Posterior tergogonocoxal muscle; Stylet retractor muscle	http://purl.obolibrary.org/obo/HAO_0001616

## Abbreviations

1vf: 1st valvifer
1vv: 1st valvula
2vf: 2nd valvifer
2vv: 2nd valvula
3vv: 3rd valvula
ba: Basal articulation
af9: Anterior flange of T9
au: Aulax
ba: Basal articulation
blb: Bulb
ca: Cordate apodeme
cls: Comb-like scales
co: Common oviduct
ct: Ctenidia
ct1: Ctenidia-like structures at the dorsal ramus
df2: Dorsal flange of the 2nd valvifer
dn1: Distal notch of the 1st valvula
dp2: Dorsal projection of the 2nd valvifer
dr1: Dorsal ramus of the 1st valvula
ec: Egg canal
gm: Genital membrane
hsl: Hook-shaped lobe of the 2nd valvifer
iar: Interarticular ridge of the 1st valvifer
il1: Interlock of the 1st valvulae
iva: Intervalvifer articulation
lb: Laminated bridge
lu1: Lumen of 1st valvulae
m-1vf-gm: 1st valvifer-genital membrane muscle
m-a-2vf-2vv: Anterior 2nd valvifer-2nd valvula muscle
m-d-2vf-vr: Dorsal 2nd valvifer-venom gland reservoir muscle
m-d-T9-2vf-a: Dorsal T9-2nd valvifer muscle (part a)
m-d-T9-2vf-b: Dorsal T9-2nd valvifer muscle (part b)
m-p-2vf-2vv: Posterior 2nd valvifer-2nd valvula muscle
m-p-T9-2vf: Posterior T9-2nd valvifer muscle
m-T9-gm: T9-genital membrane muscle
m-v-2vf-vr-a: Ventral 2nd valvifer-venom gland reservoir muscle (part a)
m-v-2vf-vr-b: Ventral 2nd valvifer-venom gland reservoir muscle (part b)
m-v-T9-2vf: Ventral T9-2nd valvifer muscle
mb2: Median bridge of the 2nd valvifers
ml: Membranous layer
ovr: Orifice of the venom gland reservoir
pra: Processus articularis
prm: Processus musculares
rh: Rhachis
se: Sensilla
SEM: Scanning electron microscopy
sk: Stalk
sp: Sensillar patch of the 2nd valvifer
sr: Sensillar row of the 2nd valvifer
SR-µCT: Synchrotron X-ray phase-contrast microtomography
st: Sawteeth
T9: Female T9 (9th abdominal tergum)
trb: Terebra
tva: Tergovalvifer articulation
vr: Venom gland reservoir of the 2nd valvifer

## References

[1] Peters RS, Krogmann L, Mayer C, Donath A, Gunkel S, Meusemann K, et al. Evolutionary history of the Hymenoptera. Curr Biol. 2017;27:1013–8. 10.1016/j.cub.2017.01.027. 28343967 10.1016/j.cub.2017.01.027

[2] Blaimer BB, Santos BF, Cruaud A, Gates MW, Kula RR, Mikó I, et al. Key innovations and the diversification of Hymenoptera. Nat Commun. 2023;14: 1212. 10.1038/s41467-023-36868-4. 36869077 10.1038/s41467-023-36868-4 PMC9984522

[3] Vilhelmsen L, Miko I, Krogmann L. Beyond the wasp-waist: structural diversity and phylogenetic significance of the mesosoma in apocritan wasps (Insecta: Hymenoptera). Zool J Linn Soc. 2010;159:22–194. 10.1111/j.1096-3642.2009.00576.x.

[4] Quicke DLJ, LeRelac A, Vilhelmsen L. Ovipositor structure and function in the parasitic Hymenoptera with an exploration of new hypotheses. Rendiconti. 2000;47:197–239.

[5] Quicke DLJ. The braconid and ichneumonid parasitoid wasps: biology, systematics, evolution and ecology. Chichester: Wiley-Blackwell; 2015.

[6] Snodgrass RE. Morphology of the insect abdomen. Part II. The genital ducts and the ovipositor. Smithsonian Misc Collect. 1933;89:1–148.

[7] Quicke DLJ. Parasitic wasps. London: Chapman & Hall; 1997.

[8] Quicke DLJ, Fitton MG, Tunstead JR, Ingram SN, Gaitens PV. Ovipositor structure and relationships within the Hymenoptera, with special reference to the Ichneumonoidea. J Nat Hist. 1994;28:635–82. 10.1080/00222939400770301.

[9] LeRalec A, Rabasse JM, Wajnberg E. Comparative morphology of the ovipositor of some parasitic Hymenoptera in relation to characteristics of their hosts. Can Entomol. 1996;128:413–33. 10.4039/Ent128413-3.

[10] Oeser R. Vergleichend-morphologische Untersuchungen über den Ovipositor der Hymenopteren. Mitt Zool Mus Berlin. 1961;37:3–119. 10.1002/mmnz.19610370102.

[11] Heraty JM. Parasitoid biodiversity and insect pest management. In: Foottit RG, Adler PH, editors. Insect biodiversity: science and society. 2nd ed. Oxford: Wiley-Blackwell; 2017. p. 603–25. 10.1002/9781118945568.ch19.

[12] Cruaud A, Rasplus J, Zhang J, Burks R, Delvare G, Fusu L, et al. The Chalcidoidea bush of life: evolutionary history of a massive radiation of minute wasps. Cladistics. 2023. 10.1111/cla.12561. 37919831 10.1111/cla.12561

[13] Peters RS, Niehuis O, Gunkel S, Bläser M, Mayer C, Podsiadlowski L, et al. Transcriptome sequence-based phylogeny of chalcidoid wasps (Hymenoptera: Chalcidoidea) reveals a history of rapid radiations, convergence, and evolutionary success. Mol Phylogenet Evol. 2018;120:286–96. 10.1016/j.ympev.2017.12.005. 29247847 10.1016/j.ympev.2017.12.005

[14] Smith EL. Evolutionary morphology of the external insect genitalia. 2. Hymenoptera. Ann Entomol Soc Am. 1970;63(1):1–27. 10.1093/aesa/63.1.1.

[15] Smith EL. Biosystematics and morphology of Symphyta—III external genitalia of *Euura* (Hymenoptera: Tenthredinidae): sclerites, sensilla, musculature, development and oviposition behavior. Int J Insect Morphol Embryol. 1972;1:321–65. 10.1016/0020-7322(72)90016-5.

[16] Vilhelmsen L. The ovipositor apparatus of basal Hymenoptera (Insecta): phylogenetic implications and functional morphology. Zool Scr. 2000;29:319–45. 10.1046/j.1463-6409.2000.00046.x.

[17] Copland MJW. Female reproductive system of the Aphelinidae (Hymenoptera: Chalcidoidea). Int J Insect Morphol Embryol. 1976;5:151–66. 10.1016/0020-7322(76)90001-5.

[18] Copland MJW, King PE. The structure of the female reproductive system in the Torymidae (Hymenoptera: Chalcidoidea). Trans R Entomol Soc Lond. 1972. 10.1111/j.1365-2311.1972.tb00363.x.

[19] Copland MJW, King PE. The structure and possible function of the reproductive system in some Eulophidae and Tetracampidae. Entomologist. 1971;104:4–28.

[20] Copland MJW, King PE. The structure of the female reproductive system in the Eurytomidae (Chalcidoidea: Hymenoptera). J Zool. 1972;166:185–212. 10.1111/j.1469-7998.1972.tb04085.x.

[21] Copland MJW, King PE. The structure of the female reproductive system in the Chalcididae (Hymenoptera). Entomol Mon Mag. 1971;107:230–9.

[22] Copland MJW, King PE. The structure of the female reproductive system in the Pteromalidae (Chalcidoidea: Hymenoptera). Entomologist. 1972;124:191–212.

[23] King PE, Copland MJW. The structure of the female reproductive system in the Mymaridae (Chalcidoidea: Hymenoptera). J Nat Hist. 1969;3(3):349–65. 10.1080/00222936900770311.

[24] King PE. The muscular structure of the ovipositor and its mode of function in *Nasonia vitripennis* (Walker) (Hymenoptera: Pteromalidae). Proc R Entomol Soc Lond Ser A Gen Entomol. 1962;37:121–8. 10.1111/j.1365-3032.1962.tb00002.x.

[25] Copland MJW, King PE, Hill DS. The structure of the female reproductiiye system in the Agaonidae (Chalcidoidea, Hymenoptera). J Entomol Ser A Gen Entomol. 1973;48:25–35. 10.1111/j.1365-3032.1973.tb00029.x.

[26] Hanna AD. The male and the female genitalia and the biology of *Eughalcidia caryobori* Hanna (Hymenoptera, Chalcidinae). Trans R Entomol Soc Lond. 1934;82:107–36. 10.1111/j.1365-2311.1934.tb00030.x.

[27] James HC. The anatomy of a British phytophagous chalcidoid of the genus *Harmolita* (*Isosoma*). Proc Zool Soc Lond. 1926;96:75–182. 10.1111/j.1096-3642.1926.tb01540.x.

[28] Bucher GE. The anatomy of *Monodontomerus dentipes* Boh., an entomophagous chalcid. Can J Res. 1948;26(d):230–81. 10.1139/cjr48d-020.

[29] Niedermayer S, Steidle JLM. The Hohenheimer Box – a new way to rear and release *Lariophagus distinguendus* to control stored product pest insects. Biol Control. 2013;64:263–9. 10.1016/j.biocontrol.2012.12.005.

[30] Niedermayer S, Pollmann M, Steidle J. *Lariophagus distinguendus* (Hymenoptera: Pteromalidae) (Förster) — past, present, and future: the history of a biological control method using *L. distinguendus* against different storage pests. Insects. 2016;7(3): 39. 10.3390/insects7030039. 27490572 10.3390/insects7030039 PMC5039552

[31] Haye T, Olfert O, Weiss R, Mason PG, Gibson G, Gariepy TD, et al. Bioclimatic analyses of *Trichomalus perfectus* and *Mesopolobus morys* (Hymenoptera: Pteromalidae) distributions, two potential biological control agents of the cabbage seedpod weevil in North America. Biol Control. 2018;124:30–9. 10.1016/j.biocontrol.2018.06.003.

[32] Eggs B, Fischer S, Csader M, Mikó I, Rack A, Betz O. Terebra steering in chalcidoid wasps. Front Zool. 2023;20:26. 10.1186/s12983-023-00503-1. 37553687 10.1186/s12983-023-00503-1 PMC10408236

[33] Lewis KA, Tzilivakis J, Warner DJ, Green A. An international database for pesticide risk assessments and management. Hum Ecol Risk Assess. 2016;22:1050–64. 10.1080/10807039.2015.1133242.

[34] Bartlett BR, Lagace CF. A new biological race of *Microterys flavus* introduced into California for the control of *lecaniine* coccids, with an analysis of its behavior in host selection. Ann Entomol Soc Am. 1961;54(2):222–7. 10.1093/aesa/54.2.222.

[35] Hart WG. Compensatory releases of *Microterys flavus* as a biological control agent against brown soft Scale. Environ Entomol. 1972;1:414–9. 10.1093/ee/1.4.414.

[36] Ceballos M, Hernández M. *Microterys flavus* (Howard) (Chalcidoidea: Encyrtidae) as bioregulator of *Coccus hesperidum L.* and *Ceroplastes floridensis* Comst. (Homoptera: Coccidae) for Cuba. Rev Prot Veg. 1991;6:75–6.

[37] Yoder MJ, Mikó I, Seltmann KC, Bertone MA, Deans AR. A gross anatomy ontology for Hymenoptera. PLoS ONE. 2010;5: e15991. 10.1371/journal.pone.0015991. 21209921 10.1371/journal.pone.0015991 PMC3012123

[38] Seltmann KC, Yoder MJ, Mikó I, Forshage M, Bertone MA, Agosti D, et al. A hymenopterists’ guide to the Hymenoptera Anatomy Ontology: utility, clarification, and future directions. J Hymenopt Res. 2012;27:67–88.

[39] Hymenoptera Anatomy Consortium. Hymenoptera Anatomy Ontology. 2022. http://glossary.hymao.org. Accessed 10 December 2023.

[40] Fulton BB. Notes on *Habrocytus cerealellae*, parasite of the angoumois grain moth. Ann Entomol Soc Am. 1933;26:536–53. 10.1093/aesa/26.4.536.

[41] Ratcliffe NA, King PE. The “venom” system of *Nasonia vitripennis* (Walker) (Hymenoptera: Pteromalidae). Proc R Entomol Soc Lond Ser A Gen Entomol. 1967;42:49–61. 10.1111/j.1365-3032.1967.tb01002.x.

[42] Billen JPJ. The Dufour gland closing apparatus in *Formica sanguinea* Latreille (Hymenoptera, Formicidae). Zoomorphology. 1982;99:235–44. 10.1007/BF00312297.

[43] Billen J. Morphology and ultrastructure of the Dufour’s and venom gland in the ant *Myrmecia gulosa* (Fabr.) (Hymenoptera, Formicidae). Aust J Zool. 1990;38: 305. 10.1071/ZO9900305.

[44] Lieberman ZE, Billen J, van de Kamp T, Boudinot BE. The ant abdomen: the skeletomuscular and soft tissue anatomy of *Amblyopone australis* workers (Hymenoptera: Formicidae). J Morphol. 2022;283:693–770. 10.1002/jmor.21471. 35373404 10.1002/jmor.21471

[45] Ernst AF, Mikó I, Deans AR. Morphology and function of the ovipositor mechanism in Ceraphronoidea (Hymenoptera, Apocrita). J Hymenopt Res. 2013;33:25–61. 10.3897/JHR.33.5204.

[46] Frühauf PE. Legeapparat und Eiablage bei Gallwespen (Cynipidae). Z Wiss Zool. 1924;121:656–723.

[47] Sampalla B, Eggs B, Betz O. Bending the sting: joint-free movement principles in the ovipositor of the parasitoid wasp *Leptopilina heterotoma* (Figitidae). Mitt Dtsch Ges allg angew Entomol. 2018;21:171–4.

[48] van Meer NMME, Cerkvenik U, Schlepütz CM, van Leeuwen JL, Gussekloo SWS. The ovipositor actuation mechanism of a parasitic wasp and its functional implications. J Anat. 2020;237:689–703. 10.1111/joa.13216. 32533567 10.1111/joa.13216 PMC7495304

[49] Csader M, Mayer K, Betz O, Fischer S, Eggs B. Ovipositor of the braconid wasp *Habrobracon hebetor*: structural and functional aspects. J Hymenopt Res. 2021;83:73–99. 10.3897/jhr.83.64018.

[50] Eggs B, Birkhold AI, Röhrle O, Betz O. Structure and function of the musculoskeletal ovipositor system of an ichneumonid wasp. BMC Zool. 2018;3:12. 10.1186/s40850-018-0037-2.

[51] Mehrnejad MR, Copland MJW. Behavioral responses of the parasitoid *Psyllaephagus pistaciae* (Hymenoptera: Encyrtidae) to host plant volatiles and honeydew. Entomol Sci. 2006;9:31–7. 10.1111/j.1479-8298.2006.00151.x.

[52] van Baaren J, Nénon J. Host location and discrimination mediated through olfactory stimuli in two species of Encyrtidae. Entomol Exp Appl. 1996;81:61–9. 10.1111/j.1570-7458.1996.tb02015.x.

[53] Rosenheim JA, Rosen D. Influence of egg load and host size on host-feeding behaviour of the parasitoid *Aphytis lingnanensis*. Ecol Entomol. 1992;17:263–72.

[54] Bartlett BR, Medved RA. The biology and effectiveness of *Diversinervus elegans* (Encyrtidae: Hymenoptera), an imported parasite of lecaniine scale insects in California. Ann Entomol Soc Am. 1966;59:974–6. 10.1093/aesa/59.5.974.

[55] Weseloh RM. Sense organs of the hyperparasite *Cheiloneurus noxius* (Hymenoptera: Encyrtidae) important in host selection processes. Ann Entomol Soc Am. 1972;65(1):41–6. 10.1093/aesa/65.1.41.

[56] Cerkvenik U, van de Straat B, Gussekloo SWS, van Leeuwen JL. Mechanisms of ovipositor insertion and steering of a parasitic wasp. Proc Natl Acad Sci USA. 2017. 10.1073/pnas.1706162114. 28847936 10.1073/pnas.1706162114 PMC5604017

[57] Cerkvenik U, Dodou D, van Leeuwen JL, Gussekloo SWS. Functional principles of steerable multi-element probes in insects. Biol Rev. 2018;94:555–74. 10.1111/brv.12467. 30259619 10.1111/brv.12467 PMC7379267

[58] Vincent JFV, King MJ. The mechanism of drilling by wood wasp ovipositors. Biomimetics. 1995;3:187–201.

[59] Sakes A, Dodou D, Breedveld P. Buckling prevention strategies in nature as inspiration for improving percutaneous instruments: a review. Bioinspir Biomim. 2016;11(2): 021001. 10.1088/1748-3190/11/2/021001. 26891469 10.1088/1748-3190/11/2/021001

[60] Hodgson CJ, Henderson RC. Fauna of New Zealand Coccidae (Insecta: Hemiptera: Coccoidea). Fauna N Z. 2000.

[61] Shah ZA. Morphology, ultrastructure, and probable functions of the sense organs on the ovipositor stylets of the hymenoptran parasitoid, *Venturia canescens* (Gravenhorst). Microsc Res Tech. 2012;75:876–83. 10.1002/jemt.22007. 22223268 10.1002/jemt.22007

[62] Quicke DLJ, Fitton MG, Harris J. Ovipositor steering mechanisms in braconid wasps. J Hymenopt Res. 1995;4:110–20.

[63] Quicke DLJ. Ovipositor mechanics of the braconine wasp genus *Zaglyptogastra* and the ichneumonid genus *Pristomerus*. J Nat Hist. 1991;25:971–7. 10.1080/00222939100770631.

[64] Arakawa R. Attack on the parasitized host by a primary solitary parasitoid, *Encarsia formosa* (Hymenoptera : Aphelinidae): the second female pierces, with her ovipositor, the eggs laid by the first one. Appl Entomol Zool. 1987;22:644–5. 10.1303/aez.22.644.

[65] Netting JF, Hunter MS. Ovicide in the whitefly parasitoid, *Encarsia formosa*. Anim Behav. 2000;60:217–26. 10.1006/anbe.2000.1463. 10973724 10.1006/anbe.2000.1463

[66] Billen J, Ito F. The basicoxal gland, a new exocrine structure in poneromorph ants (Hymenoptera, Formicidae). Acta Zool. 2006;87:291–6. 10.1111/j.1463-6395.2006.00244.x.

[67] Nadein K, Gorb S. Lubrication in the joints of insects (Arthropoda: Insecta). J Zool. 2022;316:24–39. 10.1111/jzo.12922.

[68] Bartlett BR. Patterns in the host-feeding habit of adult parasitic Hymenoptera. Ann Entomol Soc Am. 1964;57:344–50. 10.1093/aesa/57.3.344.

[69] Leius K. Influence of food on fecundity and longevity of adults of *Itoplectis conquisitor* (Say) (Hymenoptera: Ichneumonidae). Can Entomol. 1961;93:771–80. 10.4039/Ent93771-9.

[70] Chan MS, Godfray HCJ. Host-feeding strategies of parasitoid wasps. Evol Ecol. 1993;7:593–604. 10.1007/BF01237823.

[71] Giron D, Rivero A, Mandon N, Darrouzet E, Casas J. The physiology of host-feeding in parasitic wasps: implications for survival. Funct Ecol. 2002;16:750–7.

[72] Quicke DLJ. The ovipositor and ovipositor sheaths. In: Quicke DLJ, editor. The braconid and ichneumonid parasitoid wasps: biology, systematics, evolution and ecology. Chichester: Wiley; 2015. p. 35–56. 10.1002/9781118907085.ch3.

[73] Dweck HKM, Gadallah NS, Darwish E. Structure and sensory equipment of the ovipositor of *Habrobracon hebetor* (Say) (Hymenoptera: Braconidae). Micron. 2008;39:1255–61. 10.1016/j.micron.2008.03.012. 18467111 10.1016/j.micron.2008.03.012

[74] Austin AD, Browning TO. A mechanism for movement of eggs along insect ovipositors. Int J Insect Morphol Embryol. 1981;10:93–108. 10.1016/S0020-7322(81)80015-3.

[75] Rahman MH, Fitton MG, Quicke DLJ. Ovipositor internal microsculpture in the Braconidae (Insecta, Hymenoptera). Zool Scr. 1998;27:319–32. 10.1111/j.1463-6409.1998.tb00464.x.

[76] Fergusson NDM. A comparative study of the structures of phylogenetic importance of female genitalia of the Cynipoidea (Hymenoptera). Syst Entomol. 1988;13:13–30. 10.1111/j.1365-3113.1988.tb00225.x.

[77] Alam S. The skeleto-muscular mechanism of *Stenobracon deesae* Cameron (Braconidae, Hymenoptera)—an ectoparasite of sugarcane and juar borers of India. Zool Ser. 1953;3:1–75.

[78] Edwards RL. The host-finding and oviposition behaviour of *Mormoniella vitripennis* (Walker) (Hym., Pteromalidae), a parasite of muscoid flies. Behaviour. 1955;7:88–111. 10.1163/156853955X00049.

[79] King PE, Rafai JA. A possible mechanism for initiating the parthenogenetic development of eggs in a parasitoid Hymenopteran, *Nasonia vitripennis* (Walker) (Pteromalidae). Entomologist. 1973;106:20–118.

[80] Wilkes A. Sperm transfer and utilization by the arrhenotokous wasp *Dahlbominus fuscipennis* (Zett.) (Hymenoptera: Eulophidae). Can Entomol. 1965;97:647–57. 10.4039/Ent97647-6.

[81] Rotheram S. Immune surface of eggs of a parasitic insect. Nature. 1967;214:700–700. 10.1038/214700a0. 6069148 10.1038/214700a0

[82] Abdalla FC, Cruz-Landim CD. Dufour glands in the hymenopterans (Apidae, Formicidae, Vespidae): a review. Rev Bras Biol. 2001;61:95–106. 10.1590/s0034-71082001000100013. 10.1590/s0034-71082001000100013 11340467

[83] van de Kamp T, Mikó I, Staniczek AH, Eggs B, Bajerlein D, Faragó T, et al. Evolution of flexible biting in hyperdiverse parasitoid wasps. Proc R Soc Lond B Biol Sci. 2022. 10.1098/rspb.2021.2086. 10.1098/rspb.2021.2086 PMC8790333 35078362

[84] Frasson L, Ferroni F, Ko SY, Dogangil G, Rodriguez y Baena F. Experimental evaluation of a novel steerable probe with a programmable bevel tip inspired by nature. J Robot Surg. 2012;6:189–97. 10.1007/s11701-011-0277-4. 27638271 10.1007/s11701-011-0277-4

[85] Nakajima K, Schwarz O. How to use the ovipositor drilling mechanism of hymenoptera for developing a surgical instrument in biomimetic design. Int J Design Nat Ecodyn. 2014;9:177–89. 10.2495/DNE-V9-N3-177-189.

[86] Leibinger A, Oldfield MJ, Rodriguez y Baena F. Minimally disruptive needle insertion: a biologically inspired solution. Interface Focus. 2016;6: 20150107. 10.1098/rsfs.2015.0107. 27274797 10.1098/rsfs.2015.0107 PMC4843620

[87] Scali M, Pusch TP, Breedveld P, Dodou D. Ovipositor-inspired steerable needle: design and preliminary experimental evaluation. Bioinspir Biomim. 2017;13: 016006. 10.1088/1748-3190/aa92b9. 29019464 10.1088/1748-3190/aa92b9

[88] de Kater EP, Sakes A, Bloemberg J, Jager DJ, Breedveld P. Design of a flexible wasp-inspired tissue transport mechanism. Front Bioeng Biotechnol. 2021. 10.3389/fbioe.2021.782037. 34858965 10.3389/fbioe.2021.782037 PMC8630668

[89] Betz O, Wegst U, Weide D, Heethoff M, Helfen L, Lee WK, et al. Imaging applications of synchrotron X-ray phase-contrast microtomography in biological morphology and biomaterials science. I. General aspects of the technique and its advantages in the analysis of millimetre-sized arthropod structure. J Microsc. 2007;227:51–71. 10.1111/j.1365-2818.2007.01785.x. 17635659 10.1111/j.1365-2818.2007.01785.x

[90] Douissard P-A, Cecilia A, Rochet X, Chapel X, Martin T, Kamp T, et al. A versatile indirect detector design for hard X-ray microimaging. J Instrum. 2012;7: P09016–P09016. 10.1088/1748-0221/7/09/P09016.

[91] Martin T, Douissard P-A, Couchaud M, Cecilia A, Baumbach T, Dupre K, et al. LSO-based single crystal film scintillator for synchrotron-based hard X-ray micro-imaging. IEEE Trans Nucl Sci. 2009;56:1412–8. 10.1109/TNS.2009.2015878.

[92] Paganin D, Mayo SC, Gureyev TE, Miller PR, Wilkins SW. Simultaneous phase and amplitude extraction from a single defocused image of a homogeneous object. J Microsc. 2002;206:33–40. 12000561 10.1046/j.1365-2818.2002.01010.x

[93] Weitkamp T, Haas D, Wegrzynek D, Rack A. ANKAphase: software for singledistance phase retrieval from inline X-ray phase-contrast radiographs. J Synchrotron Radiat. 2011;18:617–29. 21685680 10.1107/S0909049511002895

[94] Cloetens P, Pateyron-Salomé M, Buffière JY, Peix G, Baruchel J, Peyrin F, et al. Observation of microstructure and damage in materials by phase sensitive radiography and tomography. J Appl Phys. 1997;81:5878–86. 10.1063/1.364374.

[95] Cloetens P, Ludwig W, Boller E, Helfen L, Salvo L, Mache R, et al. Quantitative phase contrast tomography using coherent synchrotron radiation. In: Bonse U, editor. Proceedings of SPIE, developments in X-ray tomography III, vol. 4503. Bellingham, WA, USA: SPIE Press; 2002. p. 82–91. 10.1117/12.452867

[96] Schindelin J, Arganda-Carreras I, Frise E, Kaynig V, Longair M, Pietzsch T, et al. Fiji: an open-source platform for biological-image analysis. Nat Methods. 2012;9:676–82. 10.1038/nmeth.2019. 22743772 10.1038/nmeth.2019 PMC3855844

[97] Schneider CA, Rasband WS, Eliceiri KW. NIH image to ImageJ: 25 years of image analysis. Nat Methods. 2012;9:671–5. 10.1038/nmeth.2089. 22930834 10.1038/nmeth.2089 PMC5554542

[98] Schindelin J, Rueden CT, Hiner MC, Eliceiri KW. The ImageJ ecosystem: an open platform for biomedical image analysis. Mol Reprod Dev. 2015;82:518–29. 10.1002/mrd.22489. 26153368 10.1002/mrd.22489 PMC5428984

[99] Cardona A, Saalfeld S, Schindelin J, Arganda-Carreras I, Preibisch S, Longair M, et al. TrakEM2 software for neural circuit reconstruction. PLoS ONE. 2012;7: e38011. 10.1371/journal.pone.0038011. 22723842 10.1371/journal.pone.0038011 PMC3378562

[100] Lösel PD, van de Kamp T, Jayme A, Ershov A, Faragó T, Pichler O, et al. Introducing biomedisa as an open-source online platform for biomedical image segmentation. Nat Commun. 2020;11:5577. 10.1038/s41467-020-19303-w. 33149150 10.1038/s41467-020-19303-w PMC7642381

